# Beta cell regeneration after single-round immunological destruction in a mouse model

**DOI:** 10.1007/s00125-014-3416-4

**Published:** 2014-10-23

**Authors:** Jason M. Tonne, Toshie Sakuma, Miguel Munoz-Gomez, Moustafa El Khatib, Michael A. Barry, Yogish C. Kudva, Yasuhiro Ikeda

**Affiliations:** 1 Department of Molecular Medicine, Mayo Clinic College of Medicine, 200 First Street SW, Rochester, MN 55905 USA; 2 Department of Infectious Diseases, Mayo Clinic, Rochester, MN USA; 3 Division of Endocrinology, Mayo Clinic, Rochester, MN USA

**Keywords:** AAV vector, Beta cell proliferation, Beta cell regeneration, Prediabetes, Type 1 diabetes

## Abstract

**Aims/hypothesis:**

Achieving a better understanding of beta cell regeneration after immunological destruction is crucial for the development of immunotherapy approaches for type 1 diabetes. In previous type 1 diabetes models, sustained immune activation eliminates regenerating beta cells, thus limiting the study of the regenerative capacity of beta cells upon immunological destruction. Here, we employed an adeno-associated virus 8 (AAV8) vector for beta cell-targeted overexpression of a foreign antigen to induce single-round immunological destruction of existing beta cells.

**Methods:**

Young and aged C57BL/6J mice were treated with AAV8 vectors expressing the foreign antigen luciferase. Islet inflammation and regeneration was observed at 3, 6, 10 and 22 weeks post-AAV delivery.

**Results:**

In young C57BL/6J mice, robust humoral and cellular immune responses were developed towards antigen-expressing beta cells, leading to decreased beta cell mass. This was followed by beta cell mass replenishment, along with enhanced proliferation of insulin-positive cells, recruitment of nestin/CD34-positive endothelial cells, displacement of alpha cells and mobilisation of cytoplasmic neurogenin 3-positive cells. Mice with recovering beta cells showed normal or reduced fasting blood glucose levels and faster glucose clearance than controls. Although aged mice demonstrated similar responses to the treatment, they initially exhibited notable islet scarring and fluctuations in blood glucose levels, indicating that beta cell regeneration is slower in aged mice.

**Conclusions/interpretation:**

Our hit-and-run, beta cell-targeted antigen expression system provides an opportunity to monitor the impact of single-round immunological beta cell destruction in animals with diverse genetic backgrounds or ageing status.

**Electronic supplementary material:**

The online version of this article (doi:10.1007/s00125-014-3416-4) contains peer-reviewed but unedited supplementary material, which is available to authorised users.

## Introduction

In individuals susceptible to type 1 diabetes, exposure to environmental triggers is thought to induce abnormal activation of cellular and humoral immune responses against beta cells, leading to the initiation of beta cell destruction. The continuing destruction of beta cells results in the progressive reduction of insulin-secreting capacity [1]. At the time of clinical presentation of type 1 diabetes, approximately 60–90% of the beta cells are estimated to be destroyed or dysfunctional [2]. Notably, several studies have demonstrated direct evidence of beta cell proliferation in early-onset type 1 diabetes [3, 4]. The spontaneous recovery of beta cell function in some patients with early-onset type 1 diabetes also suggests that beta cells retain their regenerative capacity in type 1 diabetes [5]. Nevertheless, primarily because of the technical difficulties involved in monitoring beta cell mass in type 1 diabetic patients, the capacity for beta cell regeneration following immunological beta cell destruction has not been determined.

A better understanding of beta cell regeneration during or after immunological destruction may lead to the development of a curative type 1 diabetes therapy. Existing mouse models of type 1 diabetes, such as spontaneous or adoptive transfer-induced type 1 diabetes, based on NOD mice, have been widely used to study type 1 diabetes pathogenesis. In NOD mice, an increased rate of beta cell proliferation is seen prior to a significant decline in beta cell mass, with the highest levels of proliferating beta cells (3% Ki67 positive) being found at the onset of hyperglycaemia [6, 7]. Several combination therapies can reverse type 1 diabetes in NOD mice, indicating the possible recovery of endogenous insulin production in this type 1 diabetes model [8–12]. However, sustained autoimmunity towards beta cells generally results in the rapid elimination of regenerating beta cells, presenting a major barrier to monitoring beta cell regeneration capacity in type 1 diabetes models.

In this study, we employed an adeno-associated virus 8 (AAV8) vector for beta cell targeted, transient overexpression of a foreign antigen (firefly luciferase) and induced single-round immunological destruction of existing beta cells in young and aged mouse groups. Since non-integrating AAV vector DNAs are rapidly degraded in dividing cells [13], newly regenerated beta cells (from proliferation of either beta or progenitor cells) do not express the target antigen, thus enabling extensive characterisation of beta cell regeneration in the absence of sustained beta cell destruction.

## Methods

For detailed methods, see the electronic supplementary material (ESM) Methods. A summary is given below.

### Mice

All studies were approved by the Mayo Clinic Institutional Animal Care and Use Committee. Male C57BL/6J mice were purchased from the Jackson Laboratory (Bar Harbor, Maine, USA). In vivo luciferase imaging was conducted as previously described [14]. Fasting blood glucose levels were monitored using the FreeStyle Lite Blood Glucose Monitor (Abbott Laboratories, Chicago, Illinois, USA). Glucose tolerance tests were conducted by fasting the mice for 4–5 h, followed by i.p. delivery of 2 g/kg d-glucose in a 30% PBS solution. Blood glucose was checked after 0, 30, 60, 90 and 120 min.

### Cells

293T cells were maintained in DMEM supplemented with 10% calf serum, 50 U/ml penicillin and 50 μg/ml streptomycin. Cells were cultured at 37°C with 5% CO_2_.

### Plasmids

The 1.13 kbp mouse insulin 2 promoter (mIP2) [15] was PCR-amplified from mouse genomic DNA using the primers 5′-GCCAC**ACGCGT**CCCTCCTCTTGCATTTCAAAT-3′ and 5′-TCCACA**GGATCC**TGTTGAAACAATAACCTGGAA-3′. pAAV-mIP2-Luc vectors were generated by replacing the cytomegalovirus promoter of the pAAV-CMV-Luc plasmid [14] with the mIP2 sequence (bold text indicates Mlu1 and BamH1 restriction sites). The pAAV-mIP2-emerald green fluorescent protein (EmGFP) vector was cloned by replacing the luciferase transgene with EmGFP cDNA.

### AAV8 vectors

Helper-free AAV8 vector stocks were produced as previously described [16]. The pRC-2/8 AAV8 capsid-expressing plasmid was kindly provided by J. Wilson (University of Pennsylvania, Philadelphia, PA, USA).

### AAV vector administrations

Mice received an i.p. injection of AAV8 vectors at a final dose of 2 × 10^11^ genome copies per mouse.

### Detection of anti-luciferase antibody

Luciferase-expressing and control 293T cell lysates were separated by SDS-PAGE. Plasma samples were used as the primary antibody in western blotting.

### IFN-γ ELISpot assay

The assay was performed using the IFN-*γ* ELISpot Mouse Set (BD Pharmingen, San Diego, CA, USA). Splenocytes were added to duplicate wells at a density of 0.1 × 10^6^, 0.5 × 10^6^ or 1 × 10^6^ cells per well along with DMEM-10 with or without 2.0 μg/ml ovalbumin peptide or 2.0 μg/ml firefly luciferase epitope peptide. The ovalbumin T cell-reactive peptide sequence (SIINFEKL) [17] and firefly luciferase T cell-reactive epitope (LMYRFEEEL) [18] were synthesised by GenScript (Piscataway, New Jersey, USA).

### Immunostaining

All immunostaining was conducted as previously described [16]. Antibodies and the concentrations used for immunocytochemistry are described in the ESM Methods.

### Insulitis scoring

The insulitis score was determined by following established criteria [19]. Three 7 μm thick whole head-to-tail pancreatic sections (each 200 μm in depth) were collected per animal and co-stained with anti-insulin and anti-cluster of differentiation 45 (CD45) antibodies, with DAPI labelling.

### Insulin- and glucagon-positive area analysis

Pancreatic sections were prepared and the insulin-positive area was quantified by using the formula: Percentage insulin-positive area = insulin-positive area/total tissue area × 100 [16].

### Mouse pancreatic RNA extraction

Pancreases were isolated and three tissue sections (~20 mg) were immediately processed using an RNeasy Plus Mini Kit (Qiagen, Limburg, Netherlands).

### RT-PCR and quantitative PCR

One microgram of total RNA was used to synthesise cDNA (EcoDry Premix, Clontech Laboratories, Mountain View, California, USA). Quantitative PCR was conducted using SYBR green-based expression analysis in QuantiTect Primer Assays (Qiagen). Firefly luciferase expression was determined using SYBR green quantitative PCR with primers based on a 140 bp segment of the luciferase gene: Forward FFLuc_qPCR_F, 5′-GCTATTCTGATTACACCCGAGG-3′; Reverse FFLuc_qPCR_R 5′-TCCTCTGACACATAATTCGCC-3′.

### Sample size and statistical analysis

Groups were compared by unpaired Student’s *t* test, and data are expressed as means ± SEM. Significance was set at *p* < 0.05.

## Results

### Systemic administration of AAV8 vectors containing the murine insulin 2 promoter facilitates beta cell-targeted transgene expression

**Fig. 1:**
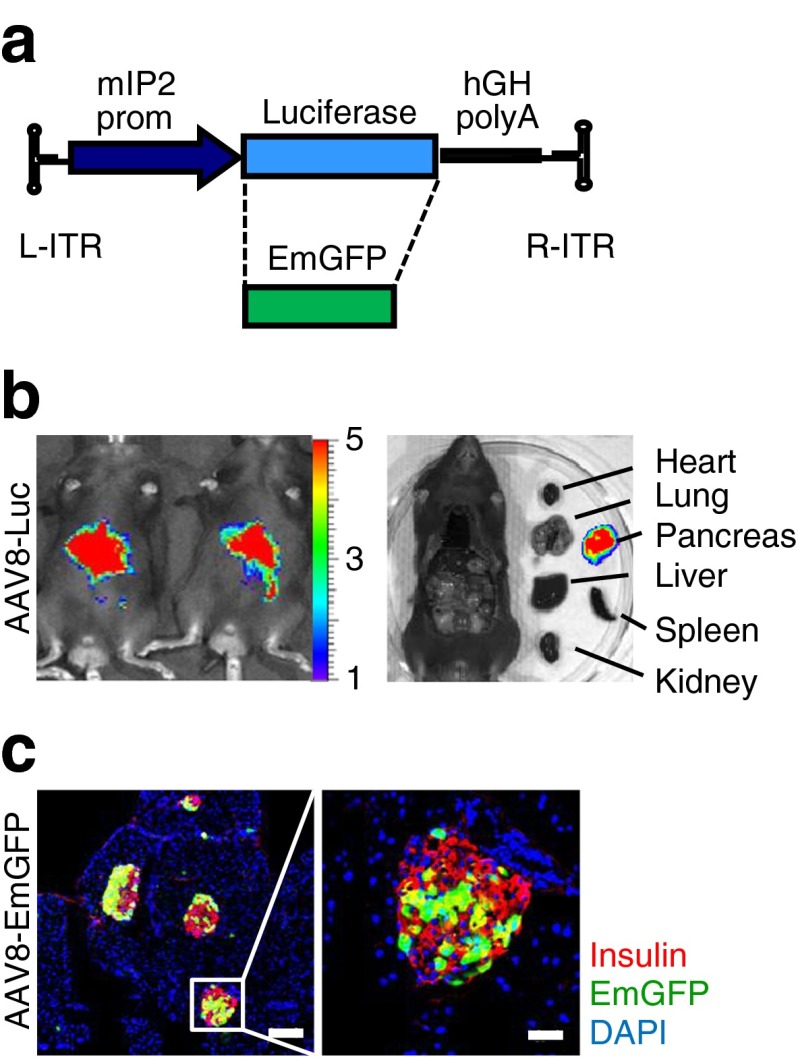
Systemic administration of AAV8 vectors containing mIP2 facilitates beta cell-targeted transgene expression. (**a**) Schematic representation of AAV vectors. L-ITR, left inverted terminal repeat; R-ITR, right inverted terminal repeat; prom, promoter. (**b**) AAV8-mediated luciferase expression was assessed 14 days after i.p. injection (control *n* = 3, AAV8-Luc *n* = 3). Luminescence scale bar, ×10^4^. (**c**) EmGFP expression was assessed in pancreatic sections EmGFP (*n* = 2). Selective EmGFP expression (green) was observed in insulin-positive beta cells (red), but not in acinar cells. Nuclei were counterstained with DAPI (blue). Scale bars, 100 μm

AAV8 vectors were engineered to express firefly luciferase under the control of an internal promoter of the mouse *Ins2* gene (Fig. 1a, ESM Fig. 1a, b). Mice were i.p. injected with the AAV8 vectors, and luciferase expression was monitored 2 weeks post infection (p.i.). AAV vectors containing mIP2 [15] exhibited potent, pancreas-restricted luciferase expression (Fig. 1b). When the mIP2-AAV8 vector was used to deliver the EmGFP gene (Fig. 1a), i.p. administration of AAV8 vectors (2 × 10^11^ genome copies/mouse) resulted in selective EmGFP expression in insulin-positive beta cells (Fig. 1c), demonstrating beta cell-specific transgene expression via the mIP2-AAV8 vector system. When EmGFP transduction efficiency was assessed from 15 random islets, the proportion of EmGFP-positive islet mass reached up to 66% (average 47.8%), relative to the insulin-positive area (*n* = 2).

### Beta cell targeted luciferase expression induces luciferase-specific humoral and cellular immune responses

**Fig. 2:**
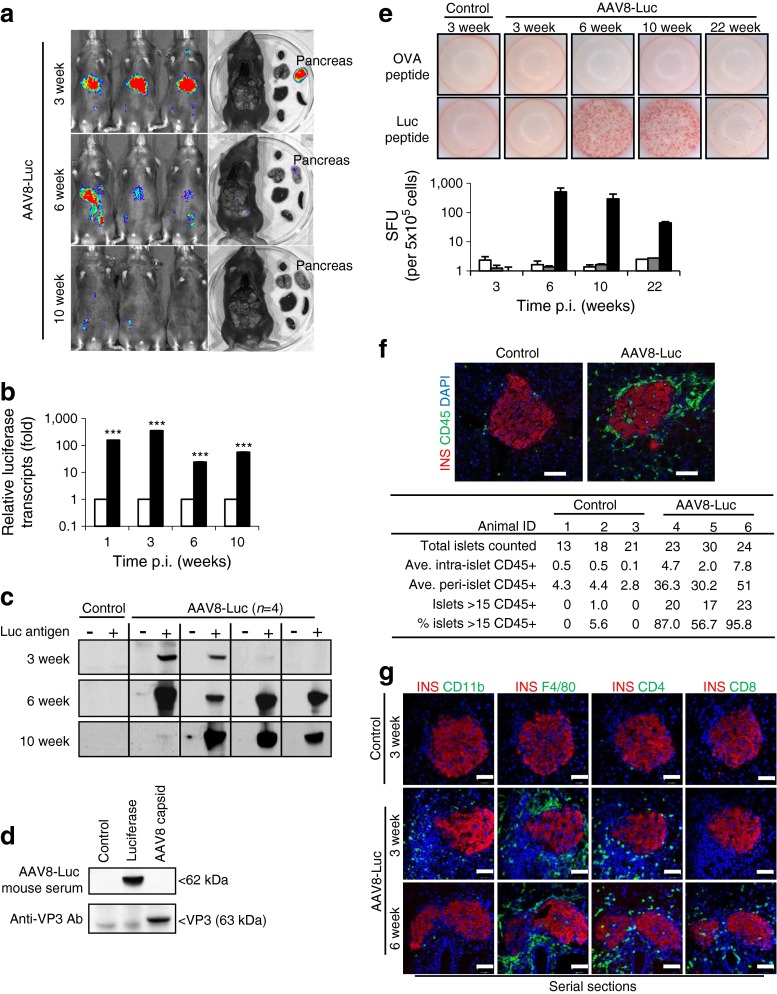
Beta cell-targeted luciferase expression induces specific humoral and cellular immune responses. (**a**) Luciferase expression was monitored 3, 6 and 10 weeks p.i. (**b**) RT-PCR analysis of total pancreatic lysates for firefly luciferase transcripts in saline controls and AAV8-Luc (white and black bars, respectively); data represents fold change vs age-matched controls set as 1; logarithmic scale, base 10; ***p < 0.001 vs controls. (**c**) Western blot detection of anti-luciferase antibodies in mouse plasma samples. (**d**) Lysates from 293T cells overexpressing luciferase and AAV8 capsid proteins and from uninfected controls were used as an antigen source to detect antibodies against AAV8 capsid. Plasma samples were from mice 6 weeks p.i. (*n* = 4). Anti-VP3 antibody was used as a control. (**e**) An ELISpot assay was performed to detect cytotoxic T cells from splenocytes of control (white bars) and AAV8-Luc (black bars) infected mice that react to the luciferase epitope peptide LMYRFEEEL. Ovalbumin (OVA) peptide was used as a negative control (grey bars). Splenocytes were analysed after 3, 6, 10 and 22 weeks. Representative wells and the means ± SEM of spot-forming units (SFU) from four mice per group per time point are shown. Logarithmic scale, log base 10. (**f**) Representative islets used for insulitis scoring with an anti-CD45 antibody at 4 weeks p.i. The table shows the number of random islets counted and the average (Ave.) intra- and peri-islet CD45 cells (*n* = 3 per group). (**g**) Serial pancreatic sections were stained with antibodies against the immune cell markers CD11b, F4/80, CD4 and CD8 (green). Nuclei were counterstained with DAPI (blue). Scale bars, 50 μm. INS, insulin; Luc, luciferase

Five-week-old mice (*n* = 4 per time point) were i.p. injected with the luciferase-expressing mIP2-AAV8 vector (AAV8-Luc). AAV8-Luc treated and control (PBS-injected) mice were monitored for luciferase expression (Fig. 2a). Luciferase signals in the pancreas were observed for 10 weeks p.i. Expression of beta cell-specific luciferase reached a peak at around 3 weeks p.i., followed by a dramatic decline in luminescent signals (Fig. 2a). This correlated well with a marked decline in luciferase transcripts in the pancreas (Fig. 2b). To investigate luciferase-specific adaptive immune responses, plasma samples and splenocytes were harvested and analysed at 3, 6, 10 and 22 weeks p.i. Three out of four mice showed weak-to-moderate levels of circulating antibodies against luciferase by 3 weeks p.i., whereas all mice showed strong humoral immunity against luciferase by 6 and 10 weeks p.i. (Fig. 2c). AAV capsid-specific antibody was not detected in serum (Fig. 2d). Beta cell-targeted luciferase overexpression also induced potent cellular immunity. An ELISpot assay revealed a luciferase-specific cytotoxic T lymphocyte (CTL) response at 6 weeks p.i. (Fig. 2e). The CTL response reached peak levels between 6 and 10 weeks p.i., and declined to intermediate levels by 22 weeks p.i. (Fig. 2e, lower panel).

High-grade islet infiltration by CD4- and CD8-positive T cells and macrophages was observed in a patient with recent-onset type 1 diabetes [20]. Immunohistochemical analysis of the leucocyte common antigen CD45 was performed to detect inflamed islets, defined as an islet containing more than 15 CD45-positive cells [19]. We found 0–5.6% and 57–96% islets with peri-insulitis or insulitis in control and AAV8-Luc-treated mice, respectively, at 4 weeks p.i. (Fig. 2f). Further analysis demonstrated that pancreatic islets were surrounded by CD11b-positive innate immune cells, murine macrophage marker (F4/80)-positive macrophages and CD4-positive helper T cells as early as 3 weeks p.i. At 6 weeks p.i., there were fewer CD11b-positive cells, but islets were infiltrated with CD8-positive CTLs, as well as F4/80- and CD4-positive cells (Fig. 2g). The timing of CTL infiltration into the islets correlated with the induction of the luciferase-targeted CTLs (Fig. 2e).

### Beta cell targeted immune response results in a transient beta cell mass decline followed by robust beta cell regeneration

**Fig. 3:**
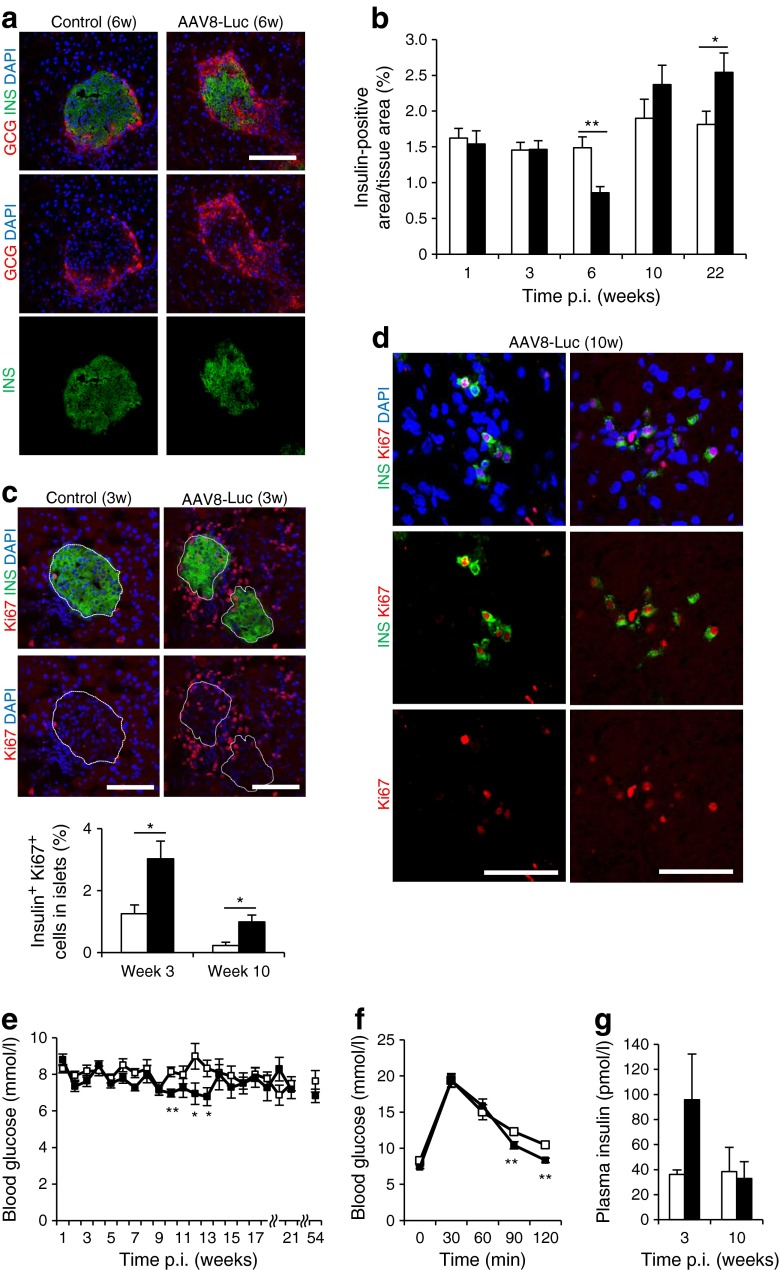
Beta cell-targeted immune responses result in a transient reduction in beta cell mass followed by robust beta cell regeneration. (**a**) Pancreatic islets were visualised by staining with anti-glucagon (GCG) and anti-insulin (INS) antibodies. Images of the islets at 6 weeks p.i. are shown. Scale bar, 100 μm. (**b**) Insulin-positive area as a percentage of the total pancreatic area in treated and control mice (*n* = 4 for each time point; *p < 0.05, **p < 0.01). (**c**) Representative pancreatic islets from AAV8-Luc treated or control mice stained with anti-insulin and anti-Ki67 antibodies. Insulin-positive and Ki67-positive islet cells from AAV8-Luc treated or control mice were counted, and the percentage of insulin/Ki67 double-positive cells was determined. Data are presented as means ± SEM. Scale bar, 100 μm. *p < 0.05 vs control. (**d**) Groups of single insulin-positive and Ki67-positive cells detected at 10 weeks p.i. are shown. Scale bars, 100 μm. (**e**) Fasting blood glucose levels were monitored (*n* = 4 per group). *p < 0.05, **p < 0.01. (**f**) Glucose tolerance test on control and treated mice (*n* = 4 per group) at 6 weeks p.i. *p < 0.05. (**g**) Fasting plasma insulin levels were determined by ELISA at 3 and 10 weeks p.i. (*n* = 3 per group per time point). (**b**), (**c**), (**e**–**g**) white bars/squares, saline controls; black bars/squares, AAV8-Luc treatment

We next measured the total insulin-positive area at multiple time points (Fig. 3a, b, ESM Fig. 2a). A significant decline in beta cell mass was observed 6 weeks post AAV delivery. The correlation between reduced beta cell mass and the induction of beta cell-targeted CTLs supports the widely accepted concept that CD8-positive CTL-mediated beta cell killing is probably a major mechanism of beta cell destruction [2]. At 10 weeks p.i., the beta cell mass recovered to normal levels. By week 22, there was a slight increase in beta cell mass in damaged islets over control (Fig. 3b).

To assess beta cell regeneration, we counted the number of insulin-positive, Ki67-positive cells within islets (Fig. 3c, upper panels). Beta cell proliferation increased in damaged and recovering islets at 3 weeks and 10 weeks p.i. by 2.4-fold (3.03%) and 4.4-fold (0.99%), respectively (Fig. 3c). In addition, AAV-Luc-treated mouse pancreases frequently showed groups of single insulin-expressing cells at 10 weeks p.i., some of which were also positive for the Ki67 proliferation marker (Fig. 3d). These observations indicate that robust beta cell regeneration occurs after immunological destruction in young mice. Although we found increased apoptotic TUNEL-positive cells in and around inflamed islets at 3 and 6 week p.i., most TUNEL-positive cells were not insulin positive (ESM Fig. 2b).

In the presence of immunological beta cell damage, AAV-Luc-transduced mice remained normoglycaemic: fasting blood glucose levels remained <10 mmol/l (Fig. 3e). The only trend we observed was a moderate decline in fasting blood glucose levels between weeks 8 and 13. This observation was also made following expression of another foreign antigen, ovalbumin, via the mIP2-AAV8 vector (ESM Fig. 3a). Body weights were the same for mice in both groups (ESM Fig. 3b). Upon glucose stimulation at 3, 6, 10 and 54 weeks post-AAV delivery, treated mice responded similarly to controls, but with slightly faster glucose clearance at 6 weeks p.i. (Fig. 3f, ESM Fig. 3c). Additionally, transient elevations in circulating insulin and glucagon-like peptide 1 (GLP-1) were observed during the immunological attack that occurred at 3 weeks p.i., but not during beta cell recovery at 10 weeks (Fig. 3g and ESM Fig. 3d).

### Displacement of glucagon-positive cells and activation of neurogenin-3-positive cells following immunological beta cell damage

**Fig. 4:**
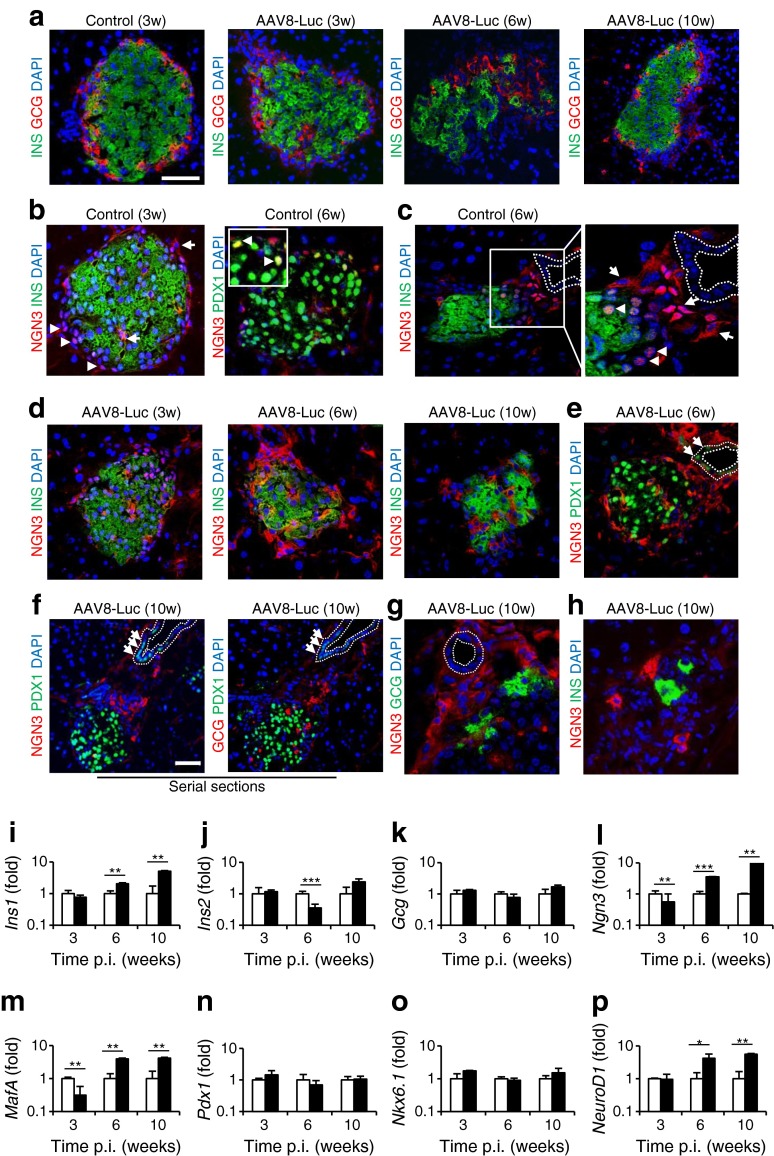
Displacement of glucagon-positive cells and activation of NGN3-positive cells following immunological beta cell damage. (**a**) The location of insulin (INS)-positive and glucagon (GCG)-positive cells was determined by staining control and immunologically damaged islets with anti-insulin (green) and anti-glucagon (red) antibodies at 3, 6 and 10 weeks p.i. (**b**) NGN3 expression was detected using an anti-NGN3 antibody in age-matched control islets at 3 and 6 weeks p.i.. Note cytoplasmic (arrows) and nuclear localisation (arrowheads) in normal islets. (**c**) Immunostaining demonstrated both nuclear (arrowheads) and cytoplasmic (arrow) NGN3 expression in age-matched control islets near ducts. (**d**) NGN3 expression was analysed in islets from AAV8-Luc-transduced mice at 3, 6 and 10 weeks p.i. Insulin expression was detected using an anti-insulin antibody. (**e**) Islets from AAV8-Luc-transduced mice at 6 weeks p.i. were analysed for NGN3 and PDX1 (green) expression. Note induction of PDX1 expression in ductal lining cells (arrows). (**f**) Representative islets showing NGN3, PDX1 and glucagon localisation after immune response. Nuclear PDX1-positive cells (arrows) were identified in ductal lining cells. (**g**) Immunostaining of the region between recovering islets and ducts with anti-NGN3 and glucagon antibodies. (**h**) A representative image of single insulin-positive cells (green) identified at 10 weeks p.i. is shown with anti-NGN3 co-staining (red). (**a**–**h**) Dashed lines correspond to ductal epithelium. Scale bars, 50 μm; w, weeks. (**i**–**p**) Quantitative RT-PCR analysis of pancreatic transcripts for common islet- and beta cell-associated factors. Results show AAV8-Luc transcripts (black bars) relative to age-matched control (white bars) pancreas, set as 1; logarithmic scale, base 10. Data are presented as means ± SEM, representing four mice per group per time point. *p < 0.05, **p < 0.01, ***p < 0.001; INS, insulin

Increased glucagon production and secretion are often associated with insulin deficiency in type 1 diabetes patients [21], while the conversion of adult alpha cells to beta cells has been reported following extreme beta cell loss [22, 23]. We assessed changes in the glucagon-expressing alpha cell population following immunological beta cell destruction. Control islets showed a normal distribution of alpha cells around the perimeter of insulin-positive islets (Fig. 4a). In contrast, following immunological beta cell injury, we frequently observed an irregular distribution or displacement of glucagon-positive cells around damaged islets (Fig. 4a, ESM Fig. 2a). The relative proportion of alpha cells was frequently increased in inflamed islets at 6 weeks p.i. (Figs 3a and 4a), with a significant increase in the glucagon-positive area relative in AAV8-Luc-treated mice (1.67 ± 0.39% and 4.70 ± 0.91% in control and treated mice, respectively). Alpha cell displacement was also evident at 10 weeks p.i., with the frequent appearance of single glucagon-positive cells outside recovering islets (Fig. 4a).

We also assessed expression of neurogenin 3 (NGN3), the beta cell progenitor marker. Nuclear NGN3 expression was evident in all control islets up to 6 weeks (up to age 12 weeks; Fig. 4b, ESM Fig. 4a). Nuclear NGN3 signals frequently co-localised with the mature beta cell marker pancreatic and duodenal homeobox 1 (PDX1) (Fig. 4b right panel, ESM Fig. 4a), while nuclear NGN3 expression was found in glucagon-positive alpha cells (ESM Fig. 4b). Strong cytoplasmic NGN3 localisation was infrequently observed in cells around control islets, especially those adjacent to pancreatic ducts (Fig. 4c, ESM Fig. 4c). These cytoplasmic NGN3-positive cells did not express insulin or PDX1.

Imaging of NGN3-positive cells in AAV vector-treated mouse pancreases showed a general decrease in nuclear NGN3 expression in beta cells and a marked increase in cytoplasmic NGN3-positive cells in and around the damaged islets (Fig. 4d, left panel), especially at 6 weeks p.i. (Fig. 4d, middle panel; ESM Figs 5 and 6). At 10 weeks p.i., cytoplasmic NGN3-positive cells were frequently found between the recovering islets and adjacent ducts (Fig. 4d, right panel, Fig. 4e, f; ESM Figs 6 and 7). This was accompanied by induction of PDX1 expression in these ductal cells (Fig. 4e, f). In contrast to control islets, glucagon-positive cells were also found at this location (between ducts and islets) at 10 weeks p.i. (Fig. 4f, ESM Fig. 7), although cytoplasmic NGN3-positive cells and glucagon-expressing cells formed distinct populations (Fig. 4g). Single insulin-positive cells were often found together with cytoplasmic NGN3-positive cells at 10 weeks p.i. (Fig. 4h).

To confirm our immunohistochemistry results, we conducted quantitative RT-PCR analysis for key pancreatic genes (Fig. 4i–p). Changes in expression of the major rodent insulin gene, *Ins2*, correlated well with changes in beta cell mass in the pancreas. Pancreatic expression of *Gcg*, *Pdx1* and *Nkx6.1* was comparable between treated and untreated mice. In contrast, expression of *Ngn3*, *MafA* and *NeuroD1*, transcription factors critical for beta cell development, was significantly elevated 6 and 10 weeks after AAV administration.

### Recruitment of NES- and CD34-positive vascular endothelial cells to damaged islets

Nestin (NES) and CD34 are key surface markers of neuronal and haematopoietic stem cells [24, 25]. Previous studies also identified NES-expressing cells as potential pancreatic progenitor cells [26]. We therefore assessed changes in NES and CD34 expression in the pancreas. Control islets consistently showed NES-positive cells within the islets and a diffuse distribution around the islet exterior (ESM Fig. 8a, left panel). During immunological beta cell damage, there was a dramatic increase in NES-positive cells around islets (ESM Fig. 8a, centre and right panels). Although pancreatic stellate cells have been reported to express NES and glial fibrillary acidic protein (GFAP) during pancreatic inflammation [27], we found NES and GFAP staining in the same location, but not necessarily in the same cells (ESM Fig. 8a, right panel). Quantitative RT-PCR analysis showed increased levels of *Nes* transcripts in inflamed pancreas at 3 weeks p.i. (ESM Fig. 8b). Recruitment of CD34-positive cells was evident at the same time point (ESM Fig. 8c). Indeed, many NES-positive cells in and around damaged islets were also positive for CD34 and endoglin (CD105; ESM Fig. 8d), suggesting that NES/CD34 double-positive cells are vascular endothelial cells. Some NES-positive cells did not express CD34 or CD105 (ESM Fig. 8d). Similar to the biased distribution of NGN3- and glucagon-positive cells during islet recovery, more NES-positive cells were found between recovering islets and ducts at 10 weeks p.i. (ESM Fig. 8e), although the NES-positive cells were distinct from glucagon- and NGN3-expressing cells (ESM Fig. 8e, f). We also induced immunological beta cell damage in a NES-positive cell lineage tracing mouse model (ESM Fig. 8g). A subset of acinar cells, as well as vascular endothelial-like cells, was labelled with membrane EmGFP (mGFP) in control and insulitis-induced mice (ESM Fig. 8g). There were no mGFP-positive beta cells, thus ruling out a direct contribution of NES-positive cells to beta cell development and regeneration.

### Induced immunological beta cell destruction leads to beta cell regeneration in aged mice

**Fig. 5:**
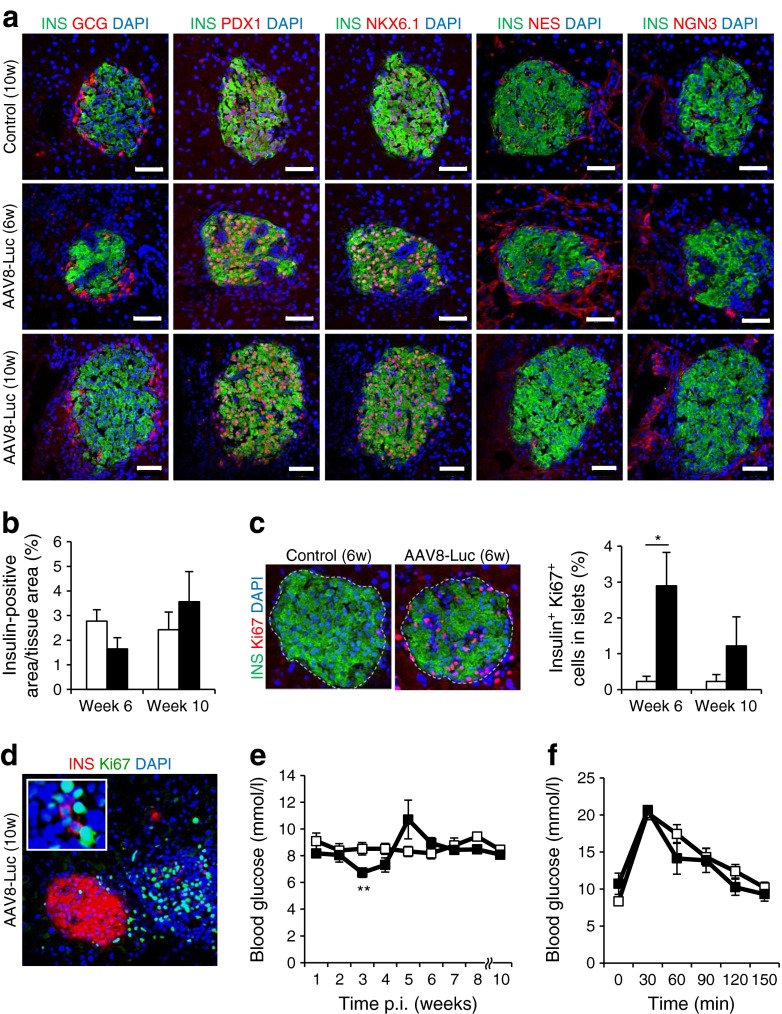
Induced immunological beta cell destruction leads to beta cell regeneration in aged mice. (**a**) Mice received AAV8-Luc vector through i.p. injection. Immunohistochemistry was performed at 6 and 10 weeks p.i. Scale bars, 50 μm. (**b**) Percentage insulin-positive area was determined as described in Fig. 3b. (**c**) Beta cell proliferation was determined as described in Fig. 3c. Images of islets at 6 weeks are shown. Beta cell proliferation was determined by quantification of total insulin- and Ki67-positive cells in five random islets from age-matched controls (*n* = 4 per time point) and AAV8-Luc-transduced mice (*n* = 4 per time point). *p < 0.05 vs control. (**d**) A representative islet in AAV8-Luc-treated mice at 10 weeks p.i., showing a dense population of Ki67-positive cells with a few isolated insulin-positive and Ki67-positive cells. (**e**) Fasting blood glucose levels were determined over the 10 week period for treated (*n* = 4) and control mice (*n* = 4). **p < 0.01 vs control. (**f**) Glucose tolerance tests were conducted at 5 weeks p.i. when vector-treated mice showed higher fasting blood glucose levels (see Fig. 5e). GCG, glucagon; INS, insulin; (**b**, **c**, **e**, **f**) white bars/squares, saline controls; black bars/squares, AAV8-Luc treatment

One-year-old mice (*n* = 6 per treatment) were i.p. injected with AAV8-Luc, and monitored for luciferase expression. Luciferase signals in the pancreas reached a peak at around 3 weeks p.i., followed by a marked decline by 10 weeks p.i. (ESM Fig. 9), which correlated with notable immune infiltration in treated islets (ESM Fig. 10a). Upon immunological beta cell destruction in aged mice, similar trends were observed in terms of induction of NES- or NGN3-positive cells (Fig. 5a, ESM Fig. 10b), reduced beta cell mass (Fig. 5b) and increased insulin-positive cell proliferation (Fig. 5c). Additionally, groups of single insulin-expressing cells, some expressing Ki67, were observed at 10 weeks p.i. (Fig. 5d, ESM Fig. 11). Although these observations demonstrate the robust regenerative capacity of beta cells following immunological damage in aged mice, many recovering islets initially exhibited notable scarring (Fig. 5a, ESM Fig. 10), and there were temporary fluctuations in blood glucose levels (Fig. 5e, f). Those observations suggest that beta cell repair responses are slower in aged mice than in their younger counterparts.

## Discussion

We used a beta cell-targeting AAV8 vector to induce single-round expression of a target antigen, firefly luciferase, in pre-existing beta cells. In this model, newly regenerated beta cells do not express the target antigen, thus allowing visualisation of beta cell recovery following immunological beta cell destruction. Luciferase expression also allowed non-invasive live imaging of beta cells under immunological attack. Since AAV vectors have broad host ranges, our ‘hit-and-run’ antigen transduction system can be used to induce immunological beta cell injury in various animal species, including rodents, large animal models and non-human primates, with particular genetic backgrounds and ageing status. It is plausible that immunological beta cell destruction in mice with a high-risk type 1 diabetes genetic background can lead to full-blown autoimmune diabetes. In addition, the use of different AAV serotype vectors, such as AAV8 and AAV9, with beta cell tropism [15, 16, 28, 29] would allow multiple rounds of vector administration to beta cells, providing an opportunity to assess the contribution of environmental triggers and the long-term influence of multiple asymptomatic immune events on beta cell proliferative capability in animal models.

Beta cell proliferation is increasingly recognised as a primary pathway for beta cell expansion during normal postnatal development, pregnancy, or following partial pancreatectomy or partial beta cell ablation [30–33]. Here, we found robust beta cell proliferation following immunological beta cell destruction in young mice. Increased beta cell proliferation was evident as early as 3 weeks p.i. (Fig. 3c, d), when luciferase-targeted humoral, but not cellular, immunity was detectable (Fig. 2e), and until replenishment of the beta cell mass at 10 weeks p.i. Although their beta cell mass recovery was slower, aged mice also demonstrated significantly increased beta cell proliferation after immunological beta cell destruction. Our data therefore suggest that beta cell proliferation is the primary mode of beta cell regeneration following immunological beta cell destruction, consistent with previous studies demonstrating increased beta cell proliferation in recent-onset type 1 diabetic patients [4, 20].

Various cell types have been proposed as potential progenitors of beta cells [34]. For instance, partial duct ligation (PDL) induces PDX1 expression in duct-lining cells and mobilises NGN3-positive cells, which can give rise to beta cells [35, 36]. The conversion of adult alpha cells to beta cells has also been demonstrated after extreme beta cell loss [22]. In our study, immune-mediated beta cell destruction frequently led to alpha cell mass increase and displacement, confirming previous studies in type 1 diabetic patients or in NOD mice with immunological beta cell damage [4, 23]. Groups of glucagon-positive cells were also found between recovering islets and adjacent ducts at 10 weeks p.i. Similarly, increased numbers of cytoplasmic NGN3-positive cells were found around damaged and recovering islets, with PDX1 induction in ductal cells near to recovering islets. At present, it is not known whether those cells can directly differentiate into beta cells. Recently, Xiao et al (2013) showed increased NGN3 expression in proliferating ductal cells and existing beta cells after PDL but that these cells do not directly contribute to beta cell neogenesis [30]. Considering that beta cell proliferation starts at 3 weeks p.i. and notable recruitment and activation of alpha cells and cytoplasmic NGN3-positive cells is initiated at later time points, it is less likely that alpha cells and cytoplasmic NGN3-positive cells play primary roles in beta cell regeneration, at least in the early stage of beta cell regeneration after immunological beta cell destruction. Since beta cell injury can activate alpha cells to produce GLP-1, a growth and survival factor for beta cells [23], it is plausible that alpha cells indirectly support beta cell recovery through a paracrine action. Combinations of genetic lineage tracing studies using our AAV8-mIP-Luc vector-mediated immunological destruction model would reveal whether specific cell types act as beta cell progenitors following immunological beta cell destruction.

NES-positive cells were initially recognised as multipotential stem cells in the adult pancreas [26]. Immunological beta cell damage induced the rapid expansion, and potentially the recruitment, of NES-positive cells in and around damaged islets. Nevertheless, our lineage tracing experiment demonstrated that NES-positive cells contribute to exocrine and microvasculature cells, but not to beta cells, supporting a previous report [37]. Our preliminary data using the AAV8-mIP2-Luc vector in NES knockout mice suggest roles for NES-positive endothelial cells in the recruitment of CD11b-positive immune cells to damaged islets and in the clearance of apoptotic beta cells from damaged islets (J. M. Tonne and Y. Ikeda, unpublished data). Further study should shed light on the role of the NES-positive cells in type 1 diabetes pathogenesis.

Another notable finding is that immunological beta cell destruction induces responses reminiscent of those reported in the PDL mouse model [35, 36], such as induction of ductal PDX1 expression, activation of NGN3-positive cells and recruitment of NES-positive cells. It is conceivable that immunological beta cell damage and physical injury to the pancreas activate a conserved beta cell recovery process.

In conclusion, we have demonstrated that the beta cell-targeted AAV8 vector can induce immunological beta cell destruction. Our results validate the use of this system as a tool to induce single-round beta cell destruction as well as visualising beta cell regeneration after immunological ablation. Our model provides a valuable platform to characterise beta cell regeneration after immunological destruction in various animal models with different genetic backgrounds and ageing status.

## Electronic supplementary material

Below is the link to the electronic supplementary material.

ESM Methods (PDF 96 kb)

ESM Fig. 1 (PDF 135 kb)

ESM Fig. 2 (PDF 140 kb)

ESM Fig. 3 (PDF 97 kb)

ESM Fig. 4 (PDF 291 kb)

ESM Fig. 5 (PDF 243 kb)

ESM Fig. 6 (PDF 268 kb)

ESM Fig. 7 (PDF 123 kb)

ESM Fig. 8 (PDF 544 kb)

ESM Fig. 9 (PDF 94 kb)

ESM Fig. 10 (PDF 388 kb)

ESM Fig. 11 (PDF 126 kb)

## Abbreviations

AAV8: Adeno-associated virus 8
AAV9: Adeno-associated virus 9
CD: Cluster of differentiation
CTL: Cytotoxic T cell
EmGFP: Emerald green fluorescent protein
F4/80: Murine macrophage marker
GFAP: Glial fibrillary acidic protein
GLP-1: Glucagon-like peptide 1
mIP2: Mouse insulin promoter 2
NES: Nestin
NGN3: Neurogenin 3
PDL: Partial ductal ligation
PDX1: Pancreatic and duodenal homeobox 1
p.i.: Post infection
