# Supplementary material for: Beta cell regeneration after single-round immunological destruction in a mouse model
Source: Diabetologia. 2014 Oct 23;58(2):313–23. doi: 10.1007/s00125-014-3416-4 (PMC4287683; doi:10.1007/s00125-014-3416-4)
Supplement: Supplementary file 1 — (PDF 96 kb) [file 125_2014_3416_MOESM1_ESM.pdf]

ESM Fig. 1

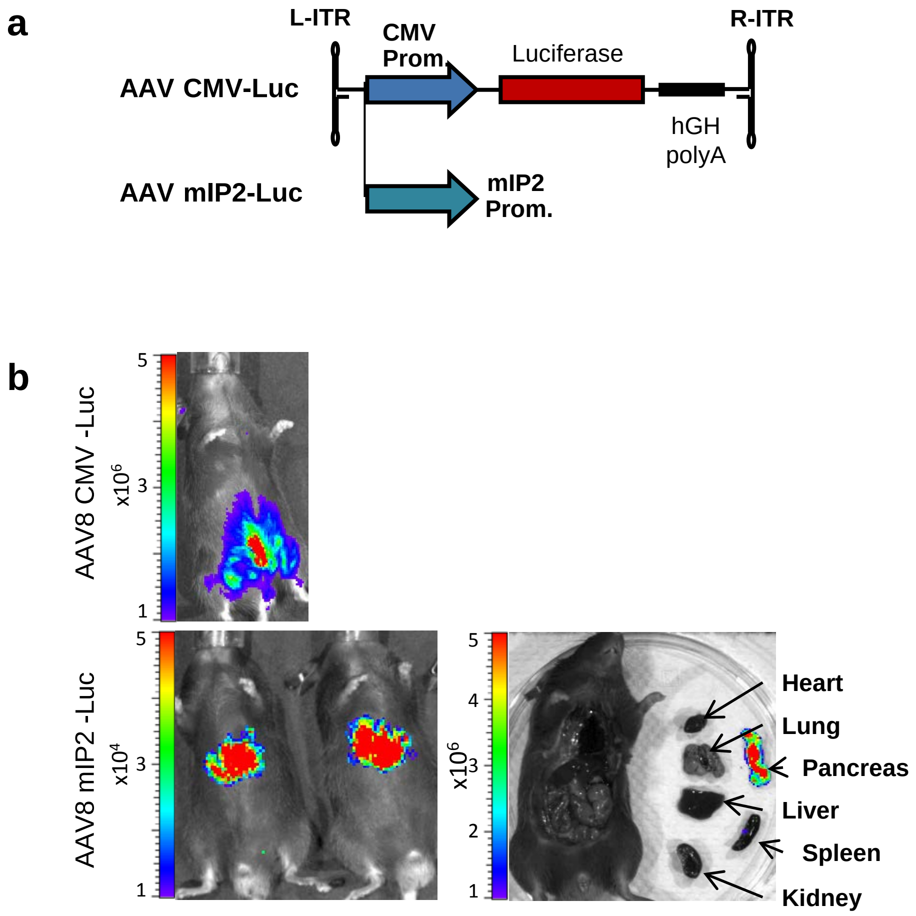

ESM Fig 1. Systemic administration of AAV8 vectors with murine insulin promoters restricts transgene expression to pancreas.

a. Schematic representation of AAV vectors with Cytomegalovirus (CMV) promoter & mouse insulin 2 gene (mIP2) promoter constructs with firefly luciferase transgenes. **b.** At 14 days after vector administration through IP, *in vivo* AAV8 mIP2-mediated luciferase expression was monitored by the IVIS live-imaging system. CMV-driven luciferase luminescence was found throughout the intraperitoneal cavity, whereas both insulin promoters restricted expression to the pancreas.
