# Supplementary material for: Beta cell regeneration after single-round immunological destruction in a mouse model
Source: Diabetologia. 2014 Oct 23;58(2):313–23. doi: 10.1007/s00125-014-3416-4 (PMC4287683; doi:10.1007/s00125-014-3416-4)
Supplement: Supplementary file 2 — (PDF 135 kb) [file 125_2014_3416_MOESM2_ESM.pdf]

ESM Fig. 2

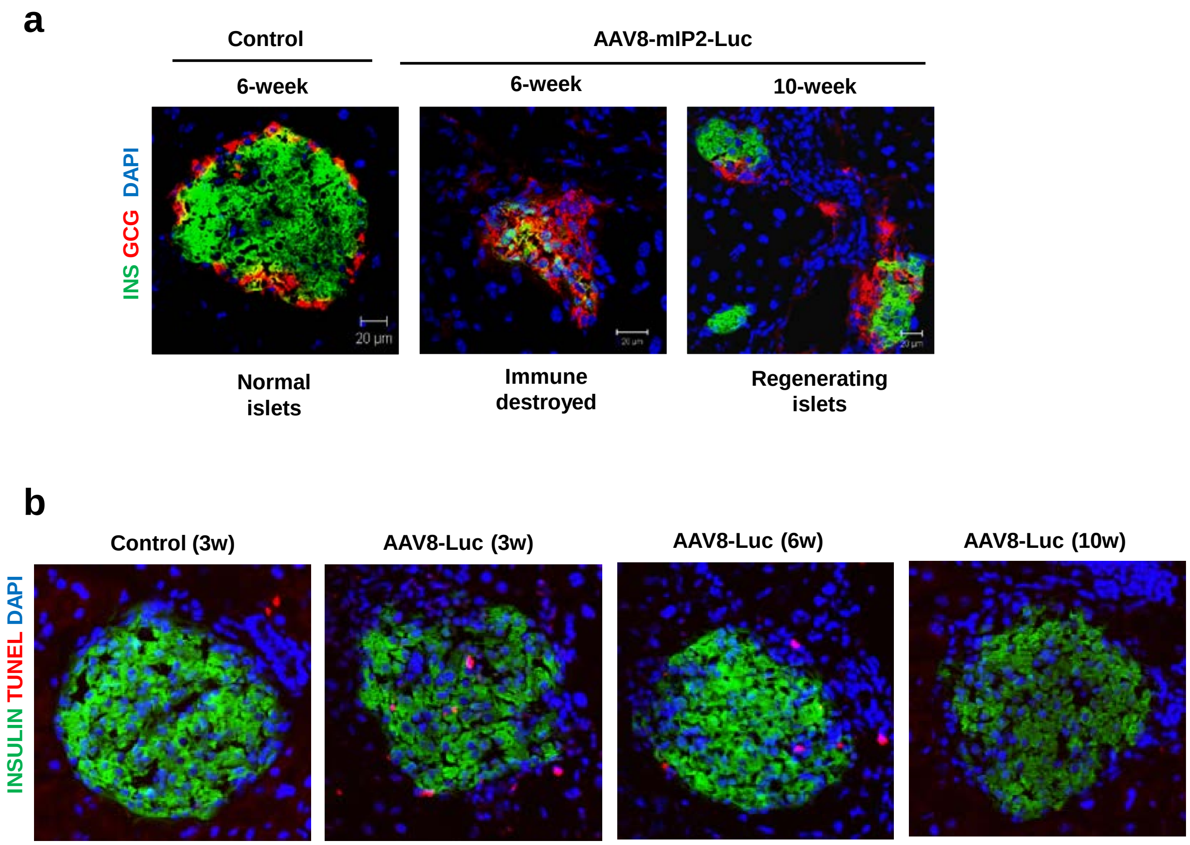

ESM Fig 2. Islet regeneration after immunological disruption.
a. Immunohistochemistry of pancreatic islet from control (left panel), immune destroyed (middle panel), and regenerating islets (right panel) **b.** TUNEL-staining for control islet and inflamed islets at 3-weeks, 6-weeks, & 10-weeks post AAV8-mIP2-Luc vector delivery.
