# Supplementary material for: Beta cell regeneration after single-round immunological destruction in a mouse model
Source: Diabetologia. 2014 Oct 23;58(2):313–23. doi: 10.1007/s00125-014-3416-4 (PMC4287683; doi:10.1007/s00125-014-3416-4)
Supplement: Supplementary file 3 — (PDF 140 kb) [file 125_2014_3416_MOESM3_ESM.pdf]

ESM Fig. 3

a. Blood glucose levels in mice treated with AAV8-mIP2-Ova

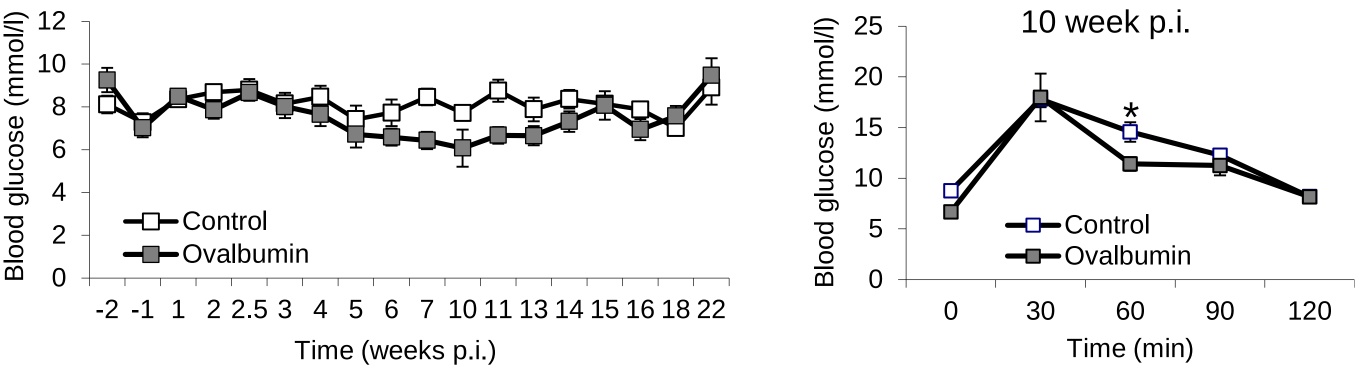

b. Body weights of AAV8 mIP2-Luc-treated and control mice

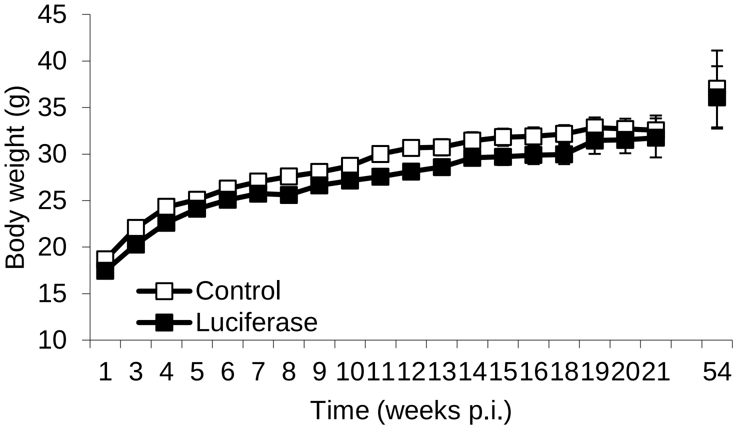

d.

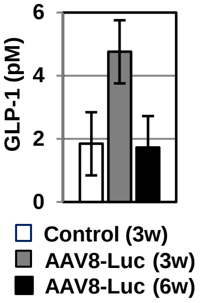

c. Glucose challenge at different time points.

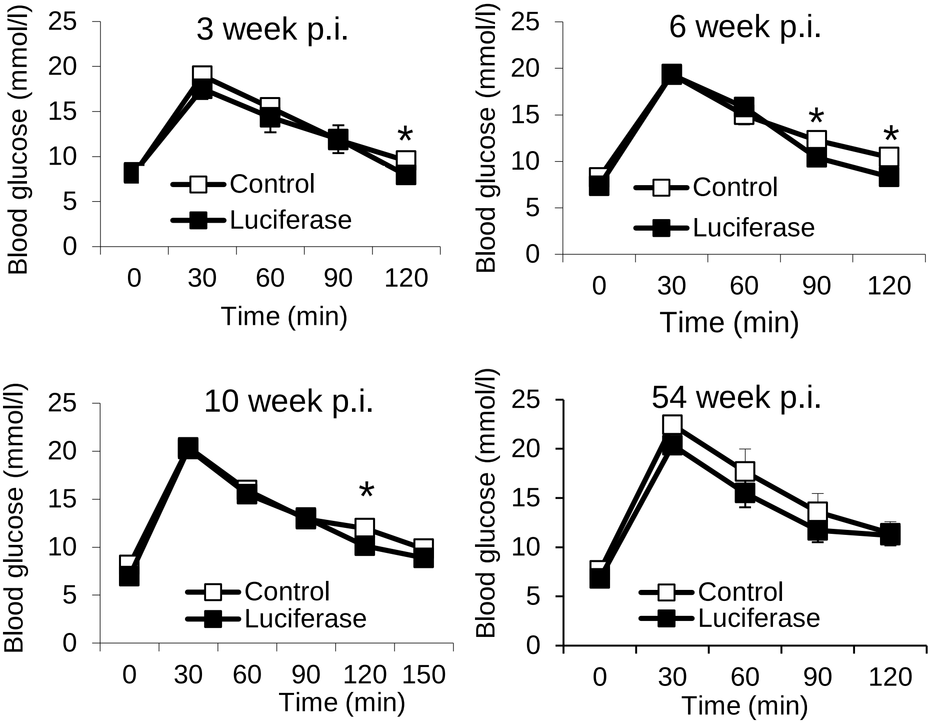

ESM Fig 3. Blood glucose dynamics from mice with immunological disrupted islets. A. Fasting blood glucose levels from mice transduced with AAV8-mIP2-Ovalbumin (n=5/group). Note lower than normal fasting blood glucose levels in the vector-treated mice at 7 to 11 weeks p.i. Glucose tolerance test performed at 10-weeks post-infection showed faster blood glucose clearance in mice treated with the Ova-expressing AAV8 vector (n=5/group, right panel). **B.** Average body weight of mice treated with the AAV8-mIP2-Luc vector over the course of 54-weeks post-infection. **C.** Glucose tolerance tests conducted at 3-, 6-, 10-, & 54-weeks post-infection with the AAV8-mIP2-Luc vector (n=4/group). **D.** Circulating GLP-1 levels from mice at 3- & 6-weeks post-infection with the AAV8-mIP2-Luc vector. *$p < 0.05$, **$p<0.001$, ***$p<0.0001$.
