# Supplementary material for: Beta cell regeneration after single-round immunological destruction in a mouse model
Source: Diabetologia. 2014 Oct 23;58(2):313–23. doi: 10.1007/s00125-014-3416-4 (PMC4287683; doi:10.1007/s00125-014-3416-4)
Supplement: Supplementary file 4 — (PDF 97 kb) [file 125_2014_3416_MOESM4_ESM.pdf]

ESM Fig. 4

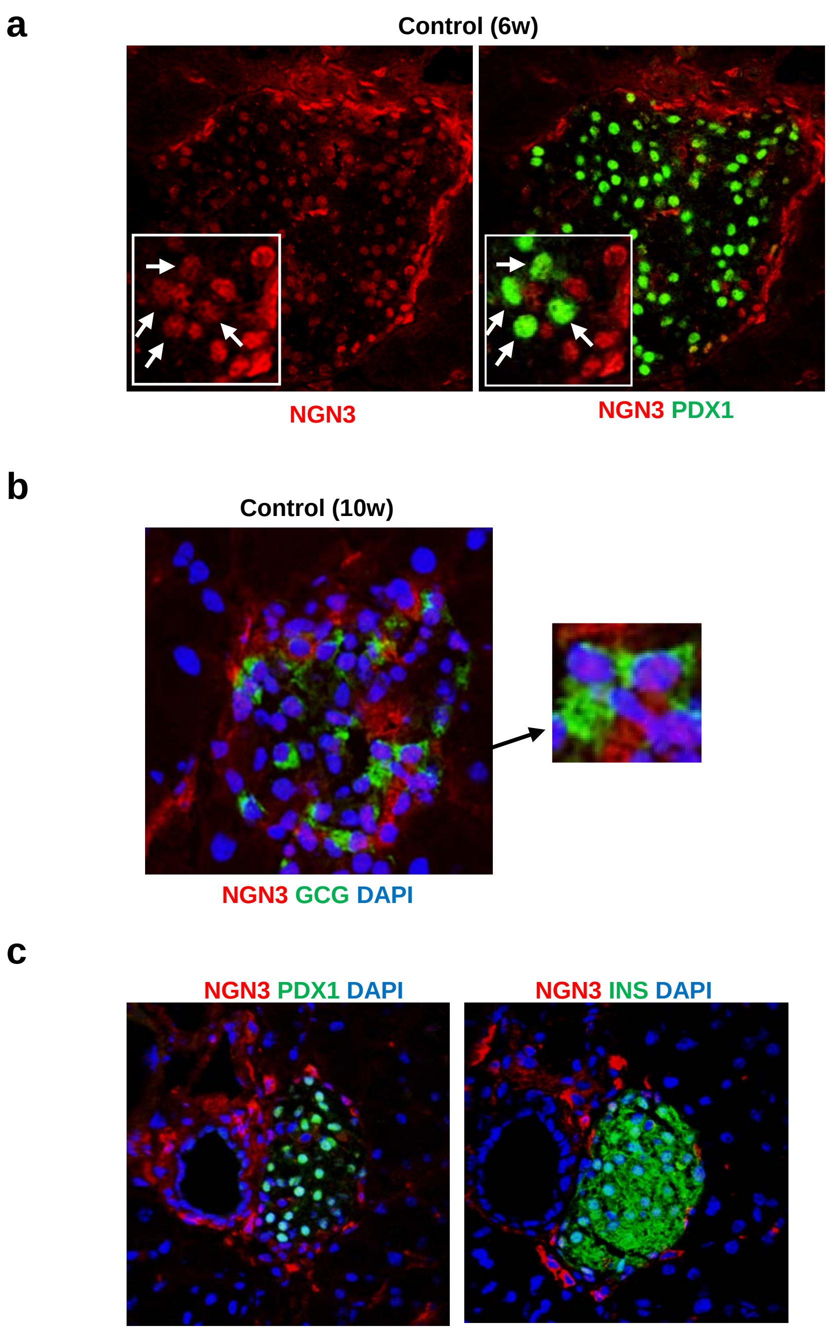

ESM Fig 4. NGN3 expression in control mice at different ages. a. Immunohistochemistry of a control pancreatic islet showing NGN3 expression and localization relative to PDX-1. Modest nuclear-localized NGN3 signals were found in PDX1-positive beta-cells, while cytoplasmic NGN3 signals were evident in cells surrounding the islet. **b.** Control pancreatic islet showing NGN3 staining relative to GCG-positive cells. Some GCG-positive alpha cells showed nuclear NGN3 signals. **c.** Control islet showing NGN3, PDX-1, and Insulin staining. In a rare occasion when islets were found adjacent to pancreatic ducts, cells with strong cytoplasmic NGN3 signals were found around the ducts and the islets.
