# Supplementary material for: Beta cell regeneration after single-round immunological destruction in a mouse model
Source: Diabetologia. 2014 Oct 23;58(2):313–23. doi: 10.1007/s00125-014-3416-4 (PMC4287683; doi:10.1007/s00125-014-3416-4)
Supplement: Supplementary file 5 — (PDF 291 kb) [file 125_2014_3416_MOESM5_ESM.pdf]

ESM Fig. 5

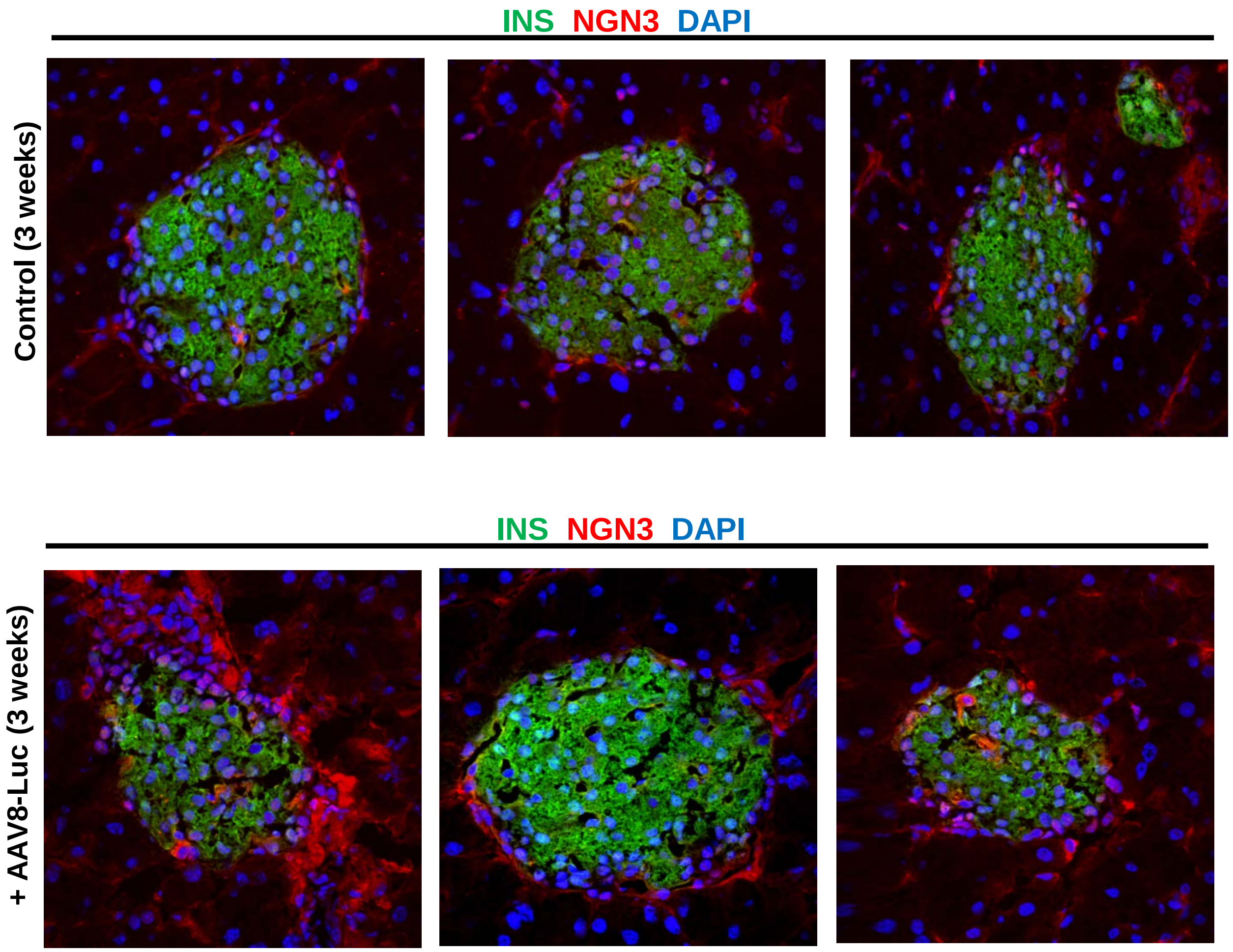

ESM Fig 5. NGN3 expression in immunological disrupted islets at 3-week post-infection. Three representative islets from three different mice at 3-weeks post-infection showing expression of NGN3 (red) within and around insulin-positive (green) islets. Images taken on 40x objective.
