# Supplementary material for: Beta cell regeneration after single-round immunological destruction in a mouse model
Source: Diabetologia. 2014 Oct 23;58(2):313–23. doi: 10.1007/s00125-014-3416-4 (PMC4287683; doi:10.1007/s00125-014-3416-4)
Supplement: Supplementary file 6 — (PDF 243 kb) [file 125_2014_3416_MOESM6_ESM.pdf]

ESM Fig. 6

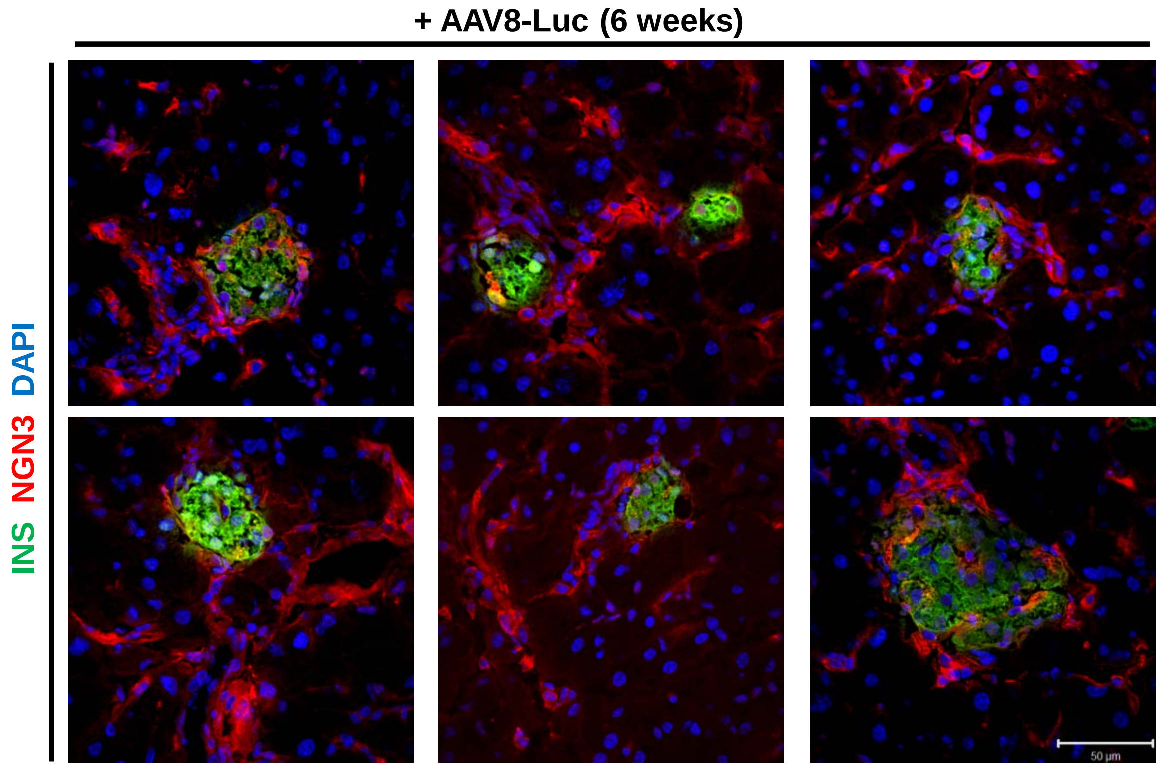

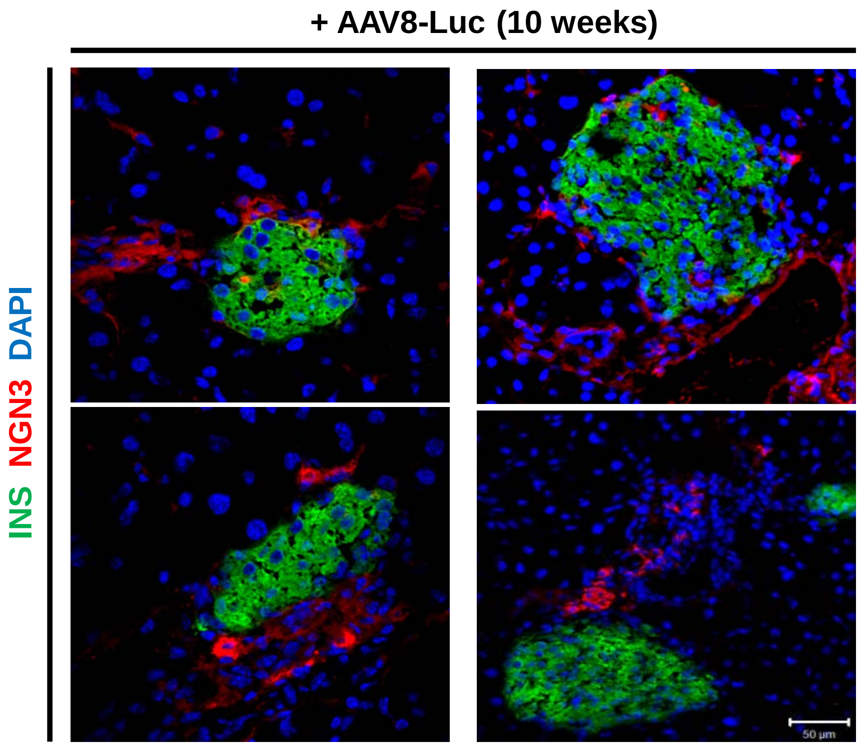

ESM Fig 6. NGN3 expression in immunological disrupted islets at 6- & 10-weeks post-infection. Representative islets from different mice at 6-weeks and 10-weeks post-infection. Both cytoplasmic and nuclear localizations of NGN3 were present in 6-week post-infected islets. By 10-week post-infection cytoplasmic NGN3 was predominant form. Images taken on 40x objective.
