# Supplementary material for: Beta cell regeneration after single-round immunological destruction in a mouse model
Source: Diabetologia. 2014 Oct 23;58(2):313–23. doi: 10.1007/s00125-014-3416-4 (PMC4287683; doi:10.1007/s00125-014-3416-4)
Supplement: Supplementary file 7 — (PDF 268 kb) [file 125_2014_3416_MOESM7_ESM.pdf]

ESM Fig. 7

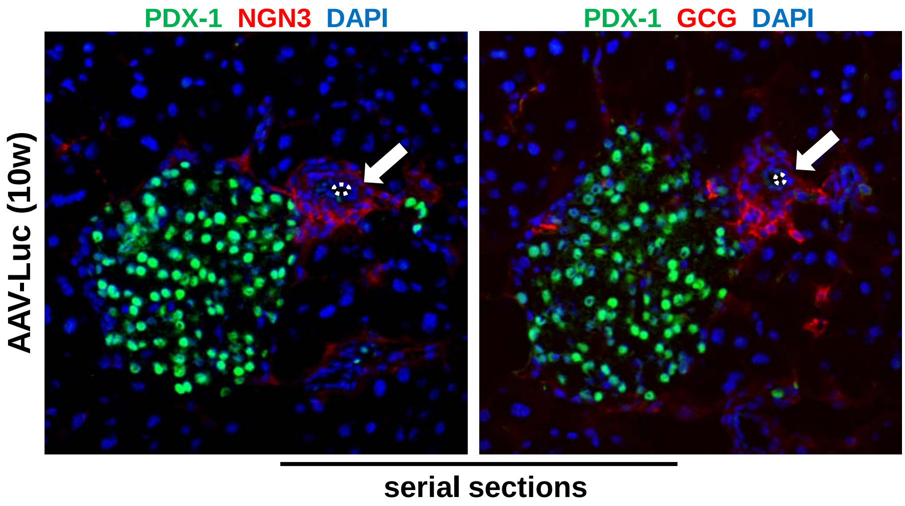

ESM Fig 7. NGN3 and GCG expression in immunologically disrupted islets at 10-weeks post-infection. Serially sectioned pancreatic islets at 10-weeks post-infection stained for PDX-1 (green), NGN3 (red, left panel) & GCG (red, right panel). Arrows indicate pancreatic duct.
