# Supplementary material for: Beta cell regeneration after single-round immunological destruction in a mouse model
Source: Diabetologia. 2014 Oct 23;58(2):313–23. doi: 10.1007/s00125-014-3416-4 (PMC4287683; doi:10.1007/s00125-014-3416-4)
Supplement: Supplementary file 8 — (PDF 123 kb) [file 125_2014_3416_MOESM8_ESM.pdf]

ESM Fig. 8

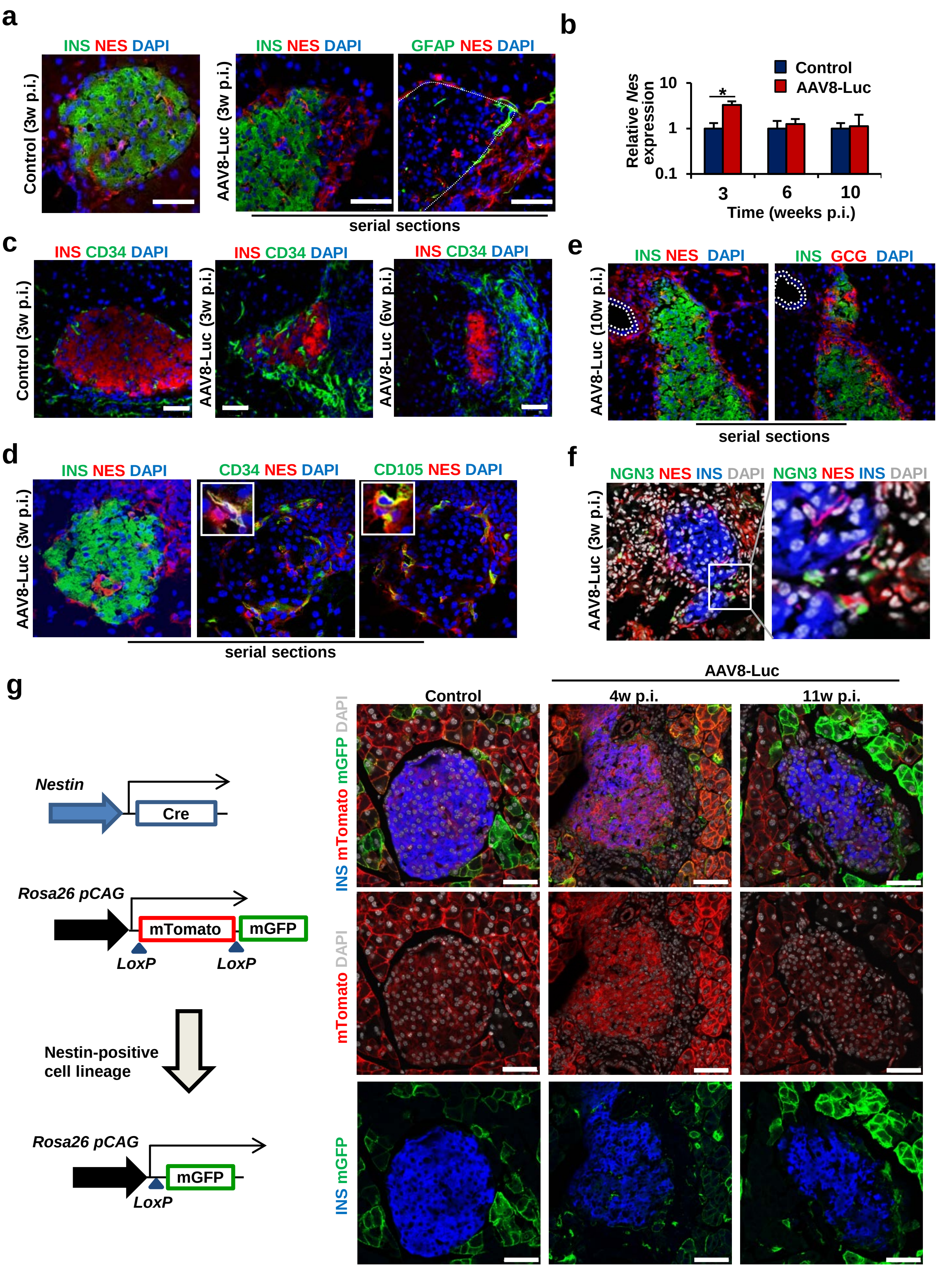

a
INS NES DAPI
Control (3w p.i.)
INS NES DAPI
GFAP NES DAPI
AAV8-Luc (3w p.i.)
serial sections
b
Control
AAV8-Luc
Relative Nes expression
10
1
0.1
*
3
6
10
Time (weeks p.i.)
c
INS CD34 DAPI
Control (3w p.i.)
INS CD34 DAPI
AAV8-Luc (3w p.i.)
INS CD34 DAPI
AAV8-Luc (6w p.i.)
e
INS NES DAPI
INS GCG DAPI
AAV8-Luc (10w p.i.)
serial sections
d
INS NES DAPI
CD34 NES DAPI
CD105 NES DAPI
AAV8-Luc (3w p.i.)
serial sections
f
NGN3 NES INS DAPI
NGN3 NES INS DAPI
AAV8-Luc (3w p.i.)
g
Nestin
Cre
Rosa26 pCAG
mTomato
mGFP
LoxP
LoxP
Nestin-positive cell lineage
Rosa26 pCAG
mGFP
LoxP
AAV8-Luc
Control
4w p.i.
11w p.i.
INS mTomato mGFP DAPI
mTomato DAPI
INS mGFP

ESM Fig. 8

ESM Fig 8. Recruitment of NES- and CD34-positive vascular endothelial cells to damaged islets. a. NES expression in the pancreas was detected by anti-NES antibody. **b.** qRT-PCR for *Nes* transcripts at 3-, 6-, and 10-wks p.i. (n=4 for each time point). *p<0.05. **c.** CD34 expression was detected by anti-CD34 antibody. **d, e.** Serial sections of the pancreatic tissue were tested for expression of NES, CD34 and CD105, or NES and GCG. **f.** Costaining with NES and NGN3 antibodies. Note NGN3-positive cells were negative for NES signals (right panel). **g.** Schematic of our NES^{Cre} $ROSA^{mTmG}$ lineage tracing mouse model. Cells with active nestin promoter generate Cre resulting in recombination of LoxP sites to activate mGFP expression. Confocal imaging showing control (left column), AAV8-Luc 4wk p.i. (middle) and AAV8-Luc 11wk p.i. (right column). **A, C and G;** Scale bars = 50 µm.
