# Supplementary material for: Beta cell regeneration after single-round immunological destruction in a mouse model
Source: Diabetologia. 2014 Oct 23;58(2):313–23. doi: 10.1007/s00125-014-3416-4 (PMC4287683; doi:10.1007/s00125-014-3416-4)
Supplement: Supplementary file 9 — (PDF 544 kb) [file 125_2014_3416_MOESM9_ESM.pdf]

ESM Fig. 9

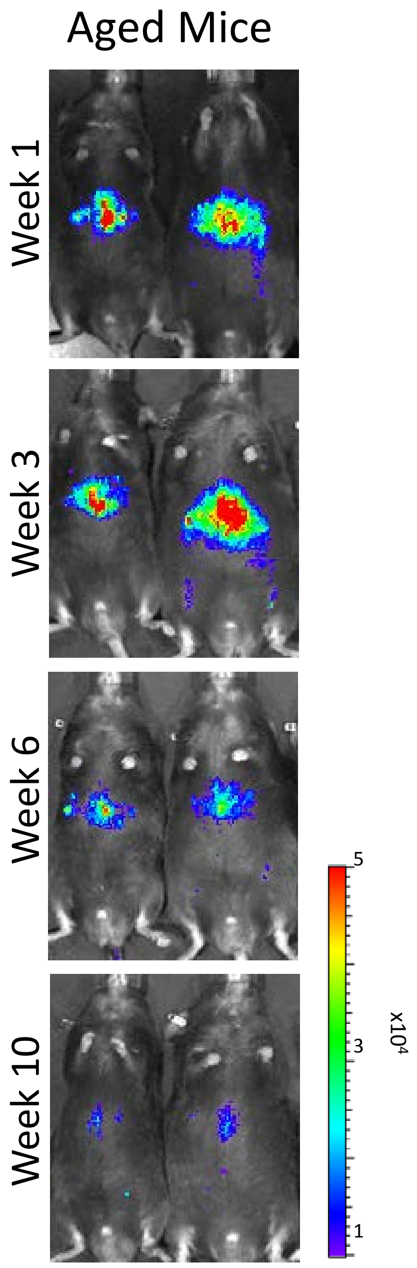

ESM Fig 9. In vivo luciferase expression in aged mice infected with the AAV8-mIP2-Luciferase vector. IVIS Xenogen imaging of same mice at 1-, 3-, 6-, 10-weeks post-infection with AAV8-mIP2-Luciferase vector is shown.
