# Supplementary material for: Beta cell regeneration after single-round immunological destruction in a mouse model
Source: Diabetologia. 2014 Oct 23;58(2):313–23. doi: 10.1007/s00125-014-3416-4 (PMC4287683; doi:10.1007/s00125-014-3416-4)
Supplement: Supplementary file 10 — (PDF 94 kb) [file 125_2014_3416_MOESM10_ESM.pdf]

ESM Fig. 10

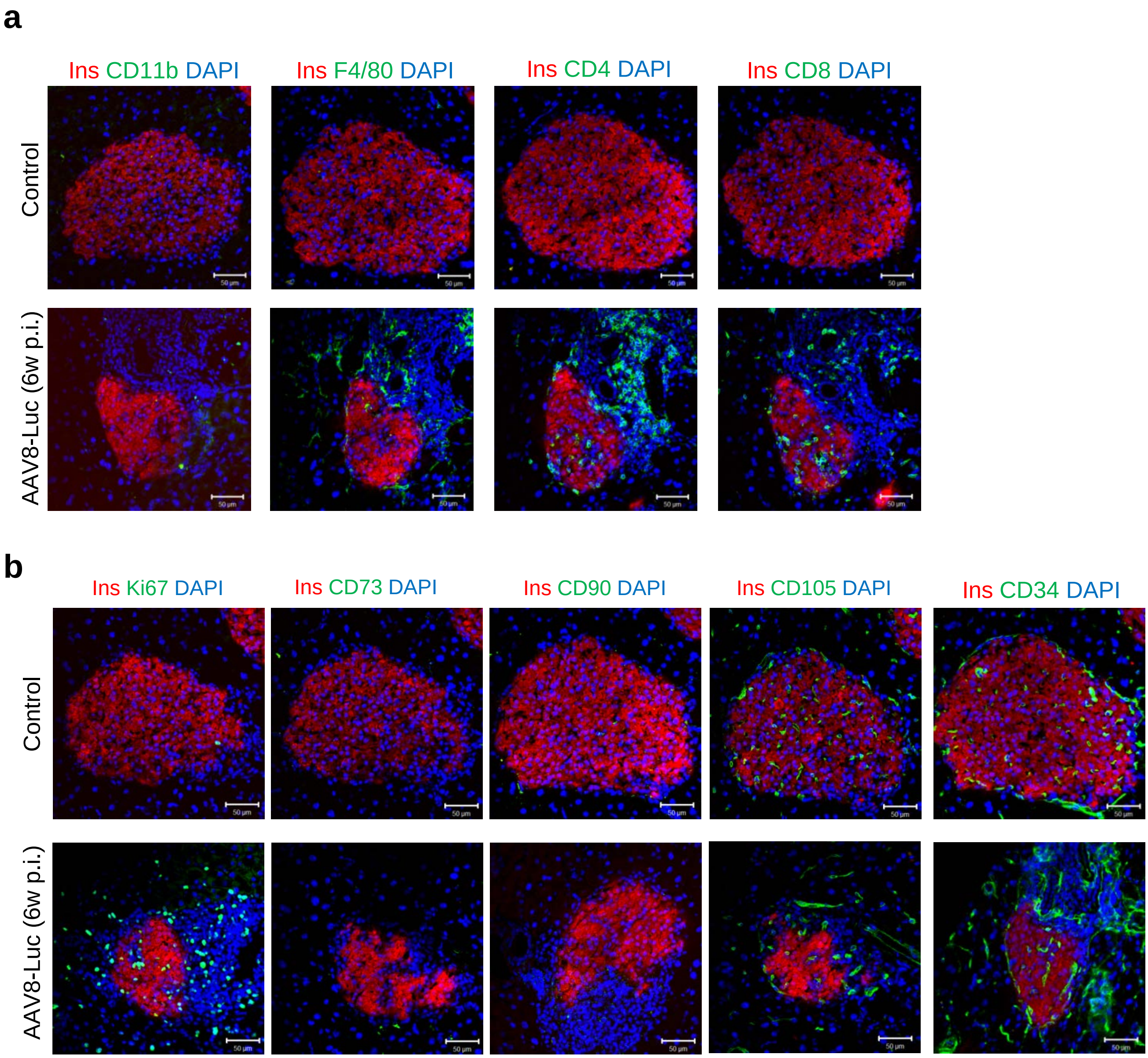

ESM Fig. 10. Pancreatic islets from aged mice overexpressing AAV8-mIP2-Luciferase. a. Immunohistochemistry was performed to determine immune infiltration in aged islets. Serial sections of the control or AAV8-mIP2-Luc vector-treated mice were stained with immune cell markers CD11b, F4/80, CD4 and CD8 antibodies (green). β-cells were visualized by anti-insulin (INS) antibody, while nuclei were counter-stained by DAPI. **b.** Immunohistochemistry of islets from aged mice (control and 6-week post-infected). Serial sections were stained with Ki67 as well as common stem cell markers CD73, CD90, CD105, and CD34. Scale bars, 50 µm.
