# Supplementary material for: Beta cell regeneration after single-round immunological destruction in a mouse model
Source: Diabetologia. 2014 Oct 23;58(2):313–23. doi: 10.1007/s00125-014-3416-4 (PMC4287683; doi:10.1007/s00125-014-3416-4)
Supplement: Supplementary file 11 — (PDF 388 kb) [file 125_2014_3416_MOESM11_ESM.pdf]

ESM Fig. 11

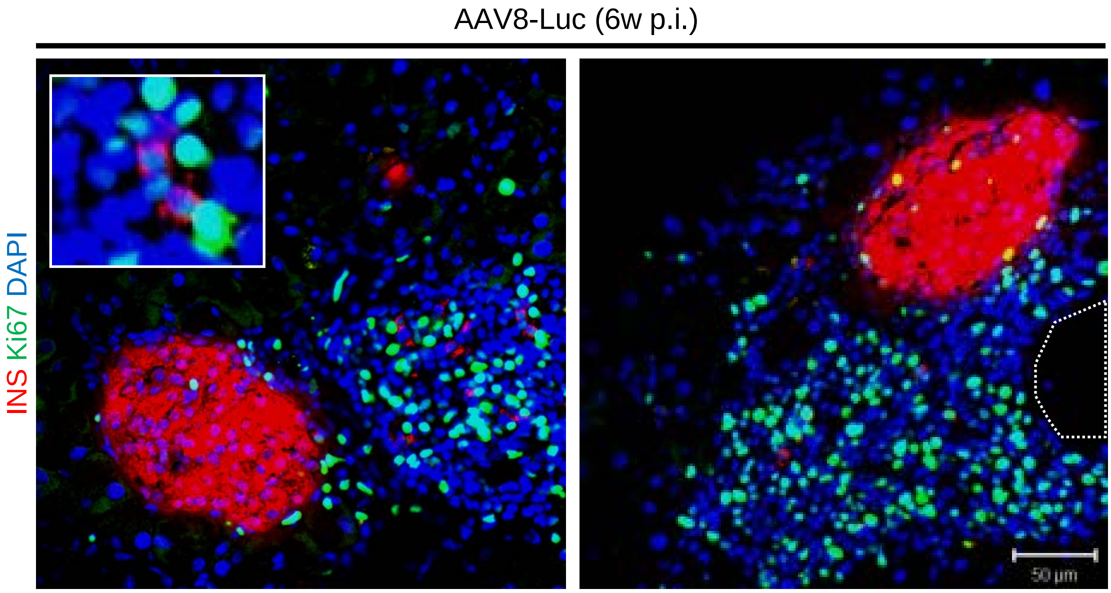

ESM Fig. 11. Immunological islet damage in aged mice show mass of proliferating cells near islets with individualized insulin-positive cells. Two representative islets from different aged AAV8-mIP2-Luc mice showing a rare occurrence of small insulin-positive cells (Red) within a mass of highly proliferative Ki67-positive (green) cells. Nuclei stained with DAPI (blue). Scale bars, 50 µm.
