# Supplementary material for: Beta cell regeneration after single-round immunological destruction in a mouse model
Source: Diabetologia. 2014 Oct 23;58(2):313–23. doi: 10.1007/s00125-014-3416-4 (PMC4287683; doi:10.1007/s00125-014-3416-4)
Supplement: Supplementary file 12 — (PDF 126 kb) [file 125_2014_3416_MOESM12_ESM.pdf]

ESM MATERIALS AND METHODS

Mice. All studies were approved by Mayo Clinic Institutional Animal Care and Use Committee. All experimental mice, C57BL/6J, nestin promoter Cre reporter (Nes^{Cre}) [1], and ROSA^{mTmG} [2] were purchased from Jackson Laboratories (The Jackson Laboratory, Bar Harbor, Maine, USA). Nestin^{Cre} and ROSA^{mTmG} mice were crossed to generate NES^{Cre} ROSA^{mTmG} strain. All mice were genotyped and only mice heterozygous for Cre and ROSA^{mTmG} were used for experiments. Mice were maintained under a 12 h light-dark cycle and were provided with irradiated Rodent Laboratory Chow (Purina 5053) and water *ad libitum*. 6-month-old mice were allowed to age additional 6-months before being used in experiments. *In vivo* IVIS luciferase imaging was conducted as previously described [3]. Fasting (4-6 h) blood glucose levels were monitored weekly or bi-weekly by FreeStyle Lite Blood Glucose Monitor (Abbott Laboratories, Chicago, Illinois, USA). Glucose tolerance tests were conducted by fasting the mice for 4-5 h followed by intraperitoneal delivery of 2g/kg of D-glucose in a 30% PBS solution. Blood glucose was checked 0, 30, 60, 90, & 120 min post-delivery.

Cells. HEK 293T cells were maintained in DMEM supplemented with 10% calf serum, 50 U/ml penicillin, and 50 µg/ml streptomycin. Cells were kept at 37°C with 5% CO2.

Plasmids. pAAV-CMV-Luc vector genome construct, which drives firefly luciferase expression by a CMV internal promoter, was described previously [3]. A 1.20 kb mouse insulin 1 promoter (mIP1) was PCR amplified from mouse genomic DNA with primers Forward Mlu1, ATCG**ACGCGT**AGAACAGGAGAGATTGAGCA and Reverse BamH1, CGAT**GGATCC**TCTCCCTAAAGTCGCTGGAGT. The 1.13 kbp mouse insulin 2 promoter (mIP2) characterized by Wang *et al.* [4] was PCR amplified from mouse genomic DNA with primers Forward Mlu1, GCCAC**ACGCGT**CCCTCCTCTTGCATTTCAAAT & Reverse BamH1, TCCACA**GGATCC**TGTTGAAACAATAACCTGGAA. pAAV-mIP1-Luc and pAAV-mIP2-Luc vectors were generated by replacing the CMV promoter of pAAV-CMV-Luc with the either of the mIP sequences by using unique MluI and BamHI restriction sites. The pAAV-mIP-EmGFP vectors were

cloned by replacing the luciferase transgene with emerald-GFP (EmGFP) by utilizing the unique restriction sites BamH1 and Xho1.

AAV8 vectors. The AAV8 vector stocks were produced in human 293T cells using the helper-free transfection method according to the manufacturer's protocol (Stratagene, Santa Clara, California, USA). Specifically, we used AAV8 capsid-expressing plasmid pRC-2/8 (kindly provided by Dr. James Wilson). Exogenous transgenes firefly luciferase- or EmGFP protein-encoding AAV genome constructs were packaged. Three days after transfection, AAV8 vector-producing 293T cells were harvested for vector purification. The cells were lysed by three freeze and thaw cycles, followed by ultracentrifuge concentration (400,000*g* for 2 h) through Optiprep Density Gradient Medium (Sigma-Aldrich, St. Louis, Missouri, USA). The resulting AAV8 vectors were desalted and concentrated using Amicon Ultra-15 100k filtration (Amicon, Billerica, Massachusetts, USA) before being resuspended in PBS. The titers (gc/ml) of concentrated AAV8 vector stocks were determined by quantitative PCR using plasmid DNA standards and the following AAV genomic sequence-specific primers and fluorescent probe: forward primer AAVhCGpA_F16, CCTGGGTTCAAGCGATTCTC, reverse primer AAVhGHpA_R17, AGCTGAGCCTGGTCATGCAT, & probe AAVhGHpA_PB TGCCTCAGCCTCCCGAGTTGTTG.

AAV8 vector administrations. Mice received an intraperitoneal injection of AAV8 vectors at a final dose of ~2e11gc/mouse.

AAV8 GFP quantification. Five-week old C57BL/6J were injected intraperitoneally with AAV8 mIP2 EmGFP vectors. Two weeks post AAV8 delivery, the mice were sacrificed and pancreas removed and immediately fixed in 4% PFA for 4-h at 4°C. After 4-h, the pancreases were then transferred to 30% sucrose/PBS solution and incubated overnight at 4°C. The next day the pancreases were transferred to 15% sucrose/PBS solution and incubated overnight at 4°C. The pancreases were then molded in O.C.T and stored at -80°C. Three 7 µm thick whole head-to-tail pancreatic sections (200 µm depths) were collected per animal and counterstained for anti-insulin with DAPI labeling. Images were collected via confocal microscopy fitted with 20x objective. GFP-positive:Insulin-positive cell mass was determined

by measuring the relative GFP-positive, Insulin-positive area to the total insulin-positive area with KS400 Image Analysis Software (Version 3.0, Zeiss, Jena, Germany).

Detection of anti-luciferase antibody. To verify the expression of neutralizing antibodies against the AAV8 delivered luciferase transgene(s), we first transfected 293T cells with either pAAV CMV Luciferase or pAAV CMV EmGFP (control) in a 6-well plate. Total cellular protein was then harvested 72 h post-transfection by first washing the cell monolayer with PBS, then coating the cells with chilled RIPA lysis buffer containing 1x complete protease inhibitor (Roche Daignostics, Indianapolis, Indiana, USA), on ice for 10 min. Cellular debris was removed by centrifugation at 10,000*g* for 10-min at 4°C. Proteins were denatured in Laemmli sample buffer containing 2-ME at 95°C for 5 min. Equal proportions of protein were loaded and separated in a 4-15% SDS-PAGE gel (BioRad Laboratories, Hercules, California, USA), before being transferred to PVDF membrane (BioRad Laboratories). Post-transfer, the resulting blots were blocked for at least 1 h in 5% milk PBS containing 0.2%Tween20. Either fresh or frozen plasma samples were then used as a primary antibody by diluting in 5% milk blocking buffer at 1:250. Antibody was allowed to incubate with blots for 14 h at 4°C. Blots were then washed 3 times in PBS with 0.05% Tween20. Anti-mouse HRP-conjugated secondary antibody (1:2000, Jackson ImmunoResearch, West Grove, Pennsylvania, USA) was diluted in 5% milk blocking buffer and incubated with blots for at least 1 h room temp, before washing 4x in wash buffer. Blots were treated with enhanced chemiluminescent substrates (Thermo Scientific, Waltham, Massachusetts, USA) and developed on x-ray films. To verify equal protein loading, each blot was treated with Restore stripping buffer (Thermo Scientific) for 15 min at 37°C, washed and reblocked in 5% blocking buffer. The blots were then incubated overnight at 4°C with Anti-β-Actin (1:2000, Sigma-Aldrich), before washing and treating with HRP-conjugated secondary (1:2000, Jackson ImmunoResearch).

IFN-γ ELISpot assay: Spleens were harvested from treated and control mice at indicated times (3 weeks, 6 weeks, 10 weeks, and 22 weeks post-AAV8 transduction) and transferred to sterile Eppendorf tubes on ice containing 2 ml of DMEM-10 (contains 10% heat inactivated FBS and 50 U/ml penicillin, and 50

µg/ml streptomycin). Once all spleens were harvested (~2-h later), the tissues were homogenized and filtered/washed through a 70 µm cell strainer using DMEM-10. Cells were centrifuged for 3 min at 800*g* at 24 °C and the resulting cell pellet was resuspended in 1 ml red blood cell lysis buffer (Sigma-Aldrich) for 10 min at room temperature, then diluted in 9-ml DMEM-10 and centrifuged for 3 min at 800*g* 24 °C. The pellet was resuspended in 2 ml DMEM-10 and viable cells were counted on hematocytometer with trypan blue. The assay was performed using the IFN-*γ* ELISpot Mouse Set (BD Pharmingen, San Diego, California, USA) according to the manufacturer's instructions. Splenocytes were added in duplicate wells at a density of either 0.1, 0.5, 1×10^6 cells per well along with no-peptide DMEM-10, 2.0µg/ml ovalbumin peptide DMEM-10, or 2.0µg/ml f. luciferase epitope peptide. Cells were incubated at 37 °C, 5% CO_2 for 16-20 h. Wells were imaged using Axiovert 40 KS400 (Zeiss), and spot forming units (SFU) were counted manually. The reported ovalbumin T-cell reactive peptide sequence (SIINFEKL [5]) and firefly luciferase T-cell reactive epitope (LMYRFEEEL [6]) was synthesized by GenScript (GenScript USA Inc, Piscataway, New Jersey, USA) .

Immuno-staining. Tissues were embedded and frozen in Optimal Cutting Temperature (O.C.T.) Compound (Sakura Finetek U.S.A., Inc., Torrance, California, USA). 7 µm pancreatic cryo-sections were obtained and immediately fixed in 4% paraformaldehyde for 30 min at room temperature. The sections were then permeabilized with PBS containing 0.3% Triton-X for 10 min, washed and then blocked for 1 h in PBS containing 5% FBS at room temperature. Primary antibody treatment was done overnight (~14 h) in humidity chambers to prevent drying. Sections were washed three times with PBS and then be incubated with species appropriate fluorescent-conjugated secondary antibody (see Table 1, ESM) for 60 min in the presence of DAPI. Cells were then observed with either Zeiss LSM 510 or Zeiss LSM 780 confocal laser scanning microscope and the images were analyzed with Zeiss imaging software. Antibodies and concentrations used for immunocytochemistry are describes in Table 1 of supplemental materials.

Insulin- and glucagon-positive area analysis. Three 7 µm thick whole head-to-tail pancreatic sections (200 µm depths) were collected per animal and counterstained for anti-insulin and anti-glucagon with DAPI labeling. Images were collected via confocal microscopy fitted with 10x objective. Insulin positive mass was determined by measuring the relative insulin-positive tissue area to total tissue area with KS400 Image Analysis Software (Version 3.0, Zeiss). Insulin positive cell area was determined using the formula: Percent Insulin Positive Area = insulin positive area/total tissue area x100. Similar analysis was performed to determine the glucagon-positive mass.

Insulitis scoring. Insulitis score was determined by following the established criteria outlined by [7]. Two 7 µm thick whole head-to-tail pancreatic sections (200 µm depths) were collected per animal and counterstained for anti-insulin and anti-CD45 with DAPI labeling. Up to 20 random islets were imaged per animal via confocal microscopy fitted with 40x objective. Peri- and intra-islet CD45-positive cells were then manually counted. Islets containing ≥15 CD45-positive cells were considered inflamed.

Mouse pancreatic RNA extraction. Murine pancreases were isolated and three tissue sections (~20 mg) were immediately processed using RNeasy Plus Mini Kit (Qiagen, Limburg, Netherlands). RNA was normalized using NanoDrop 2000 spectrophotometer (Thermo Scientific).

Reverse transcription-PCR & quantitive-PCR. RNA from pancreatic sections was isolated using the RNeasy Plus Mini Kit (Qiagen) as described above. RNA was measured and normalized. 1 ug of total polyA$^+$ RNA was then used to synthesize cDNA based on manufacture's protocol (EcoDry Premix, Clontech Laboratories, Mountain View, California, USA). Quantitive-PCR was conducted using SYBR Green-based expression analysis with QuantiTect Primer Assays from Qiagen based on manufacturer's protocol. Firefly luciferase expression was determined by SYBR green Q-PCR using primers based on a 140-bp segment of F. Luciferase's open reading frame: Forward FFLuc_qPCR_F, GCTATTCTGATTACACCCGAGG & Reverse FFLuc_qPCR_R TCCTCTGACACATAATTCGCC.

Sample size and statistical analysis. Unless specified, all groups (i.e. control and AAV8) and each time-point (i.e. 3-, 6-, 10-week etc) consisted of n=4 mice/group/time-point. All mouse experiments were

repeated at least twice. Groups were compared by unpaired Student's *t*-test, and data were expressed as mean ± SE. Significance was accepted for $p<0.05$.

Supplemental Materials & Methods

**Primary Antibodies**		
Antigen	Vendor	Dilution
Guinea Pig Anti-Insulin	Dako	1:400
Mouse Anti-Glucagon	Abcam	1:300
Rabbit Anti-Glucagon	Dako	1:100
Rabbit Anti-Pdx1	Abcam	1:100
Goat Anti-NKX6.1	R&D Sytems	1:100
Anti-Luciferase (Luc 21 1-107)	Santa Cruz	1:50
Neurogenin-3 Contributor: Madsen, O.D.	DSHB	1:100
Anti-mouse/rat Nestin	R&D Sytems	1:200
Anti-Nestin, clone rat-401, Alexa Fluor® 488 conjugate	Millpore	1:100
Rabbit Anti-Ki67	Abcam	1:100
Anti-CD11b-FITC	MACS	1:11
Rat Anti-Mouse Endoglin/CD105 (Clone 209701)	R&D Systems	1:100
Rat Anti-Thy1 CD90 RatIgG	BD Pharm	1:100
Rat Anti-Cd73 AlexaFloura 647	BD Pharm	1:100
Rat Anti-CD34	BD Pharm	1:100
Rat Anti-F4/80 Alexa Flour 488	AbD Serotec	1:100
Rat Anti-CD4	BD Pharm	1:100
Rat Anti-CD8	BD Pharm	1:100
Rabbit Anti-GFAP	Dako	1:100
Goat Anti-Ccl8	R&D Systems	1:100
Goat Anti-Cxcl9	R&D Systems	1:100
Goat Ant-Cxcl10	R&D Systems	1:100

**Secondary Antibodies**		
Species/Conjugate	Vendor	Dilution
Alexa Fluor 488 AffiniPure Donkey Anti-Guinea Pig IgG	Jackson ImmunoResearch	1:2000
Alexa Fluor 594 Goat Anti-Guinea pig IgG	Invitrogen	1:2000
Alexa Fluor 647 Donkey Anti-Guinea Pig IgG	Jackson ImmunoResearch	1:500
FITC AffiniPure Donkey Anti-mouse	Jackson ImmunoResearch	1:500
Alexa Fluor 568 Donkey Anti Mouse IgG	Invitrogen	1:2000
Alexa Fluor 488 Donkey Anti Rabbit IgG	Invitrogen	1:2000
Alexa Fluor 594 Donkey Anti Rabbit IgG	Invitrogen	1:2000
Alexa Fluor 488 Goat Anti-Rat IgG	Fisher Sci	1:2000
Alexa Fluor 494 Donkey Anti-Rat IgG	Invitrogen	1:2000
Alexa Fluor 594 AffiniPure Donkey Anti-Goat IgG	Jackson ImmunoResearch	1:2000
Alexa Fluor 594 Rabbit Anti-Goat IgG	Invitrogen	1:2000

[1] Tronche F, Kellendonk C, Kretz O, et al. (1999) Disruption of the glucocorticoid receptor gene in the nervous system results in reduced anxiety. Nat Genet 23: 99-103
[2] Muzumdar MD, Tasic B, Miyamichi K, Li L, Luo L (2007) A global double-fluorescent Cre reporter mouse. Genesis 45: 593-605
[3] Cataliotti A, Tonne JM, Bellavia D, et al. (2011) Long-term cardiac pro-B-type natriuretic peptide gene delivery prevents the development of hypertensive heart disease in spontaneously hypertensive rats. Circulation 123: 1297-1305
[4] Wang Z, Zhu T, Rehman KK, et al. (2006) Widespread and stable pancreatic gene transfer by adeno-associated virus vectors via different routes. Diabetes 55: 875-884
[5] Falk K, Rotzschke O, Faath S, et al. (1993) Both human and mouse cells expressing H-2Kb and ovalbumin process the same peptide, SIINFEKL. Cellular immunology 150: 447-452
[6] Limberis MP, Bell CL, Wilson JM (2009) Identification of the murine firefly luciferase-specific CD8 T-cell epitopes. Gene therapy 16: 441-447
[7] Campbell-Thompson ML, Atkinson MA, Butler AE, et al. (2013) The diagnosis of insulitis in human type 1 diabetes. Diabetologia 56: 2541-2543
